# Enhanced Recognition via Defect Engineered Amorphous Metal–Organic Framework for Sensitive and Stable Pesticide Biosensor

**DOI:** 10.1002/advs.202517919

**Published:** 2025-11-18

**Authors:** Changshun Su, Xiangyu Zhai, Meng Zhang, Mengxue Li, Xinru Zhang, Chunyan Sun, Xu Yan, Hongxia Li

**Affiliations:** ^1^ Department of Food Quality and Safety College of Food Science and Engineering Jilin University Changchun 130062 P. R. China; ^2^ State Key Laboratory on Integrated Optoelectronics Key Laboratory of Advanced Gas Sensors of Jilin Province College of Electronic Science & Engineering Jilin University Changchun 130012 P. R. China; ^3^ Department of Pharmacy The Second Hospital of Jilin University Changchun 130062 P. R. China

**Keywords:** amorphous MOF, biosensors, enzyme, on‐site, pesticide detection

## Abstract

Enzyme‐based biosensors with rapid and on‐site detection capabilities possess great potential for practical application. However, conventional enzyme immobilization strategies often enhance stability at the expense of substantial enzyme activity loss, thereby limiting the detection performance of enzyme‐based biosensors. Herein, a highly sensitive and robust biosensor is constructed based on defect‐engineered amorphous metal‐organic frameworks, enabling on‐site detection of organophosphate pesticides in complex food matrices. Defective acetylcholinesterase@amorphous metal‐organic frameworks (denoted as AChE@AMOF‐74) can be in situ tailored via a defect‐engineered strategy to provide a suitable microenvironment and obtain high porosity for enzyme encapsulation, while its porous architecture enhances the catalytic activity of immobilized enzyme. Impressively, the catalysis activity and target recognition ability of AChE@AMOF‐74 are 3.4‐fold and 5.6‐fold higher than those of nanoarchitectures with a regular crystalline structure. Benefiting from the structural advantages, a robust AChE@AMOF‐74‐based biosensor is constructed for the sensitive detection of pesticides, enabling quantitative analysis of paraoxon with a detection limit of 0.05 ng·mL^−1^ using an image processing algorithm. This work demonstrates the significant potential of AMOFs in constructing high‐performance enzyme‐based biosensors and has been successfully applied to detect pesticide in complex food matrices, providing a new protocol for on‐site application.

## Graphical abstract

A defect‐engineered assembly strategy is reported for the in situ encapsulation of acetylcholinesterase (AChE) into amorphous MOF‐74, endowing the composite with superior catalytic activity and target recognition capability. Leveraging these structural advantages, a sensitive and stable biosensor is constructed for the detection of paraoxon pesticide.

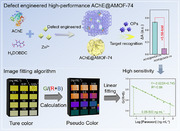

## 1 Introduction

Pesticides have been widely employed in modern agriculture due to their high efficiency and broad‐spectrum insecticidal properties.^[^ ^1^ ^]^ However, excessive or improper use of pesticides can cause persistent contamination of environment and food, and even pose serious public safety risks, underscoring the urgent need for reliable and sensitive detection methods for pesticide residues.^[^ ^2^, ^3^ ^]^ Conventional detection techniques, such as gas chromatography,^[^ ^4^ ^]^ mass spectrometry^[^ ^5^ ^]^ and capillary electrophoresis,^[^ ^6^ ^]^ offer high accuracy but often require sophisticated instrumentation, skilled operators, and time‐consuming.^[^ ^7^, ^8^ ^]^ Consequently, the development of portable, rapid, and sensitive point‐of‐care (POC) detection strategy is crucial for on‐site monitoring of pesticide residues.

Enzyme‐based POC biosensors have emerged as promising tools for the rapid and sensitive detection of pesticides due to their highly efficient target recognition and excellent signal amplification capabilities.^[^ ^9^, ^10^ ^]^ Among various enzyme systems, acetylcholinesterase (AChE) is one of the most widely employed recognition elements for the detection of organophosphate pesticides (OPs).^[^ ^11^ ^]^ It catalyzes the hydrolysis of choline esters through an acylation‐deacylation process mediated by a catalytic triad (Ser‐His‐Glu/Asp), generating thiocholine that produces a distinct colorimetric signal. ^[^ ^12^ ^]^ OPs irreversibly phosphorylate the catalytic serine residue, thereby inactivating the enzyme and suppressing thiocholine formation, which provides the basis for AChE‐based colorimetric detection.^[^ ^13^ ^]^ However, enzymes are inherently susceptible to denaturation by environmental stressors (e.g., temperature, pH, and organic solvents), which leads to reduced catalytic efficiency and poor stability, thereby significantly limiting the on‐site applications of biosensors.^[^ ^14^, ^15^, ^16^, ^17^ ^]^ Recent studies have demonstrated that various enzyme immobilization strategies effectively mitigate stability challenges under harsh conditions.^[^ ^18^, ^19^, ^20^ ^]^ Among these strategies, crystalline metal‐organic frameworks (MOFs) have attracted significant attention as promising carriers for enzyme immobilization due to their tunable pore structure, easy preparation, and excellent dispersibility.^[^ ^21^, ^22^, ^23^ ^]^ Despite the numerous advantages of MOFs, their small and highly ordered pore structures hinder effective interaction between enzyme and substrates, while also limiting the loading capacity of high‐molecular‐weight enzymes, resulting in relatively low loading efficiency (typically below 30%).^[^ ^24^, ^25^ ^]^ Additionally, the rigid framework constrains the conformational flexibility of enzymes, leading to low apparent activity of the immobilized enzymes or even complete inactivity.^[^ ^24^ ^]^ Therefore, exploring suitable scaffolds that effectively balance enzyme activity and stability is essential for the development of high‐performance POC biosensors.

Amorphous MOFs (AMOFs), which retain the structural units of crystalline MOFs and sacrifice long‐range ordered structures, have broken the tightly rigid structure of the cavity and facilitate substrate transfer, exhibiting outstanding performance in the fields of electrocatalysis and chemical sensing.^[^ ^26^, ^27^ ^]^ This unique structural arrangement expands the pore size and enhances the structural flexibility of the MOFs, while maintaining the protective function of its crystalline counterpart.^[^ ^26^ ^]^ This flexible microenvironment enables efficient encapsulation of high‐molecular‐weight enzymes, preserves their native conformation, and promotes enzyme‐substrate interactions, thereby improving catalytic performance.^[^ ^28^, ^29^, ^30^ ^]^ Compared with the crystalline MOFs, AMOFs feature a higher density of defect sites and enlarged pores, which collectively enhance its structural flexibility and porosity. These characteristics minimize enzyme distortion, improve substrate diffusion, and maintain the intrinsic activity of the encapsulated enzyme while still providing the protective microenvironment inherent to MOF frameworks.^[^ ^31^ ^]^ However, AMOFs are usually prepared by collapsing crystalline MOFs or quenching melts,^[^ ^32^ ^]^ with direct synthesis reports remaining limited. Furthermore, the disordered structure of AMOFs complicates the regulation of their morphology and performance, which remains a great challenge in the controllable regulation of AMOFs. More importantly, this limited understanding of the structural‐functional properties of AMOFs impedes their rational design, resulting in the largely unexplored potential of AMOFs with disordered and fuzzy structures as molecular hosts. Thus, there is growing interest in the possibility of hosting molecules by AMOFs with disordered and fuzzy structures.

**Scheme 1:**
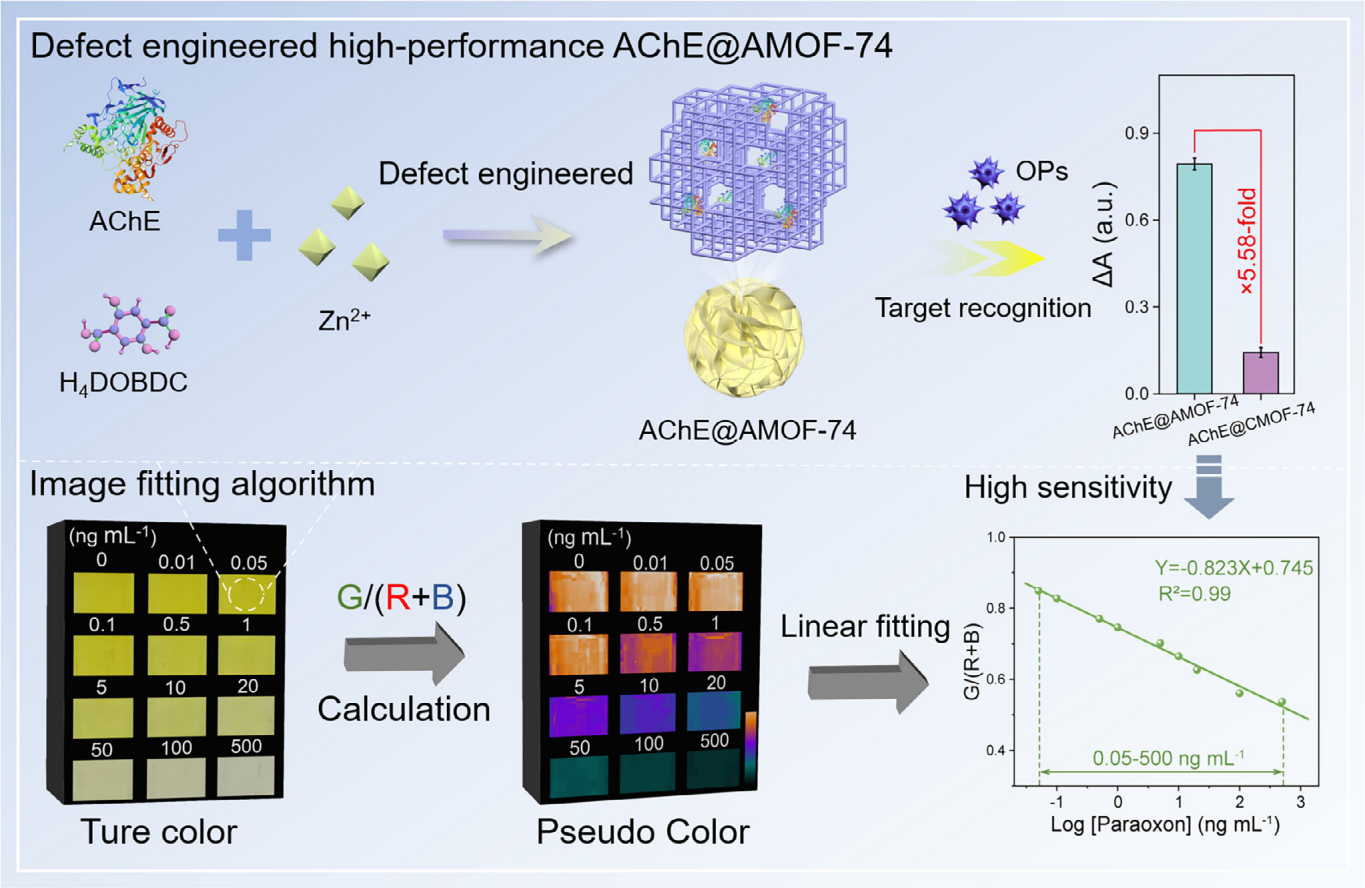
Schematic illustration of the amorphous AChE@AMOF‐74‐based biosensor for highly sensitive detection of organophosphate pesticides.

Herein, a sensitive and robust enzyme‐based POC biosensor was constructed based on defect‐engineered AChE@AMOF‐74 with high catalytic activity, enabling on‐site detection of pesticides in complex food matrices. Defective AChE@AMOF‐74 were constructed by adjusting the metal nodes‐induced competition to regulate coordination deficiency. Specifically, H_4_DOBDC functions as both the organic linker and a coordination modulator during AMOF‐74 assembly. Partial coordination competition occurs between H_4_DOBDC (25 mm) and surface residues of AChE (1 mg mL^−1^) for the Zn^2+^ (25 mm) nodes, perturbing the regular coordination of the framework. This competitive interaction generates defect sites within the MOF and facilitates the in situ encapsulation of AChE (**Scheme** **1**). Systematic studies revealed that this process introduced defect structures into the nanocomposite, thereby enlarging the pore size of the MOF and significantly enhancing the pesticide recognition capability of the sensing platform. Notably, the constructed AChE@AMOF‐74 retained 72% of its free enzyme activity, which was 3.4‐fold higher than that of nanoarchitectures with a regular crystalline structure, and AMOFs still provided effective protection for the encapsulated enzyme. Benefiting from its structural advantages, the constructed AChE@AMOF‐74‐based biosensor exhibited a pronounced colorimetric response upon exposure to paraoxon. Using an image processing algorithm to convert the colorimetric signals into digital information for precise quantification and computational correction, the detection sensitivity of the biosensor was further improved tenfold, with a detection limit as low as 0.05 ng mL^−1^. This study not only demonstrates the feasibility of customizing high‐performance enzyme@MOF composites through defect engineering, but also provides a practical and accessible approach for enhancing environmental monitoring and food safety.

## 2 Results and Discussion

### 2.1 Synthesis and Characterization of Defect‐Engineered AChE@AMOF‐74

**Figure 1:**
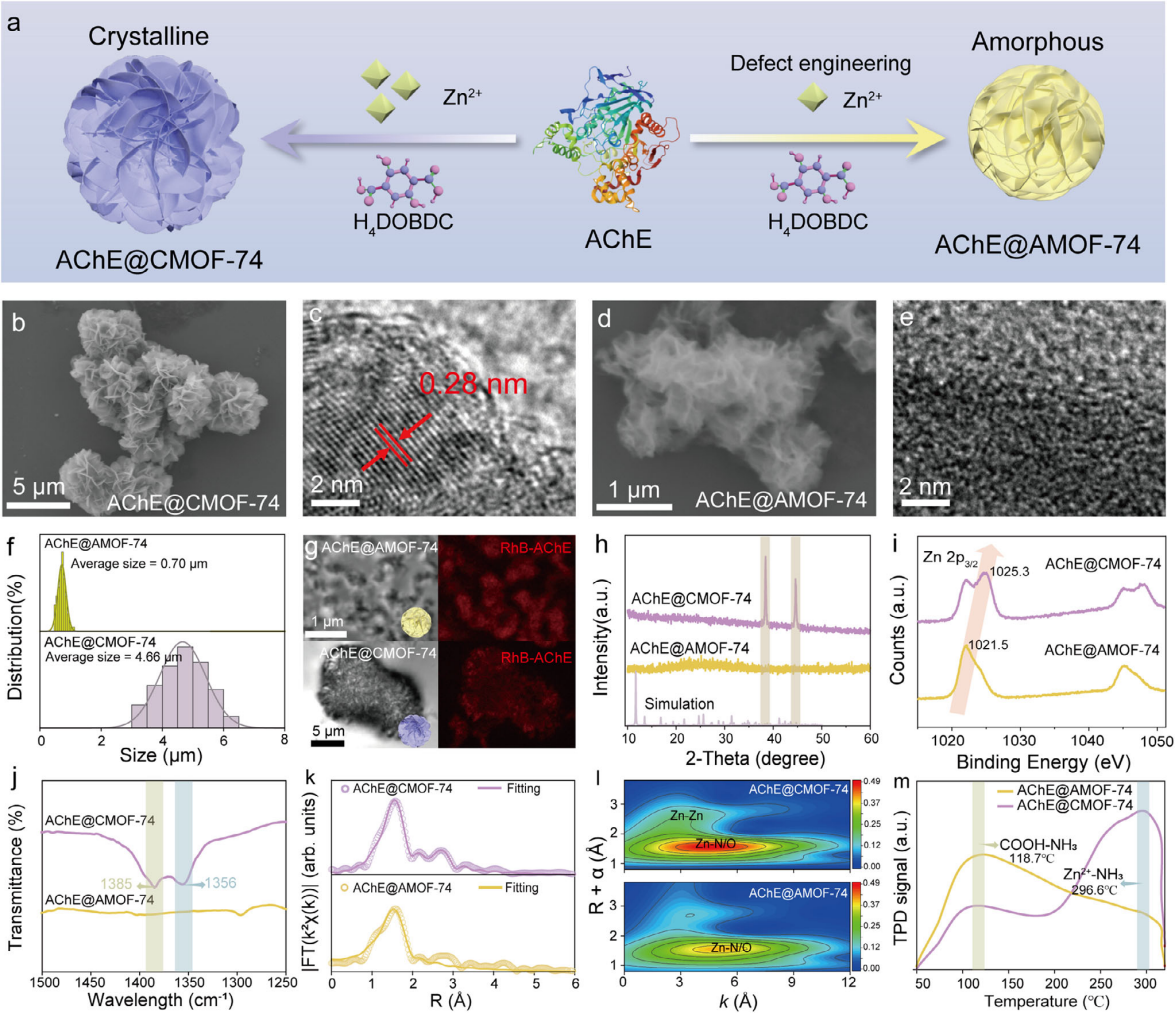
Synthesis and Characterization of Defect‐Engineered AChE@AMOF‐74. a) The synthesis diagram of AChE@AMOF‐74 and AChE@CMOF‐74. b) SEM c) high‐resolution TEM image of AChE@CMOF‐74; d) SEM e) high‐resolution TEM image of AChE@AMOF‐74; f) Size distribution of AChE@AMOF‐74 and AChE@CMOF‐74. g) Confocal fluorescence microscopy images of AChE@AMOF‐74 and AChE@CMOF‐74, in which AChE was visualized by RhB labeling (red). h) XRD spectrum of AChE@AMOF‐74 and AChE@CMOF‐74. i) High‐resolution XPS spectra of Zn 2p_3/2_ of AChE@AMOF‐74 and AChE@CMOF‐74. j) FT‐IR spectra of AChE@AMOF‐74 and AChE@CMOF‐74 in the wavelength range of 1500–1200 cm^−1^. k) Fourier transform EXAFS fitting curves of AChE@AMOF‐74 and AChE@CMOF‐74. l) Wavelet transforms of the k^2^‐weighted Zn *K*‐edge EXAFS signals of AChE@AMOF‐74 and AChE@CMOF‐74. m) NH_3_‐TPD profiles of AChE@AMOF‐74 and AChE@CMOF‐74.

Enzyme was encapsulated in conventional MOF‐74 (AChE@CMOF‐74) to obtain a highly crystalline composite (**Figure** **1**a–c). However, their catalytic activity is typically below 25% of free enzyme, making them unsuitable for the construction of a biosensor (Figure S1, Supporting Information). To address this limitation, defective AChE@AMOF‐74 were fabricated by adjusting the concentration of metal node (Zn^2+^) to trigger the competition between enzyme and ligand for regulating coordination deficiency (Figure 1a). The AChE@AMOF‐74 (Zn^2+^ = 25 mm, Zn^2+^:H_4_DOBDC = 1:1) was rapidly synthesized at 25 °C within 10 min via amorphous aggregation driven by competitive coordination between AChE and Zn^2+^ (Figures S2–S5, Supporting Information). Scanning electron microscopy (SEM) images revealed that AChE@AMOF‐74 exhibits a loosely packed flower‐core‐like structure with an average size of 0.7 µm, which is markedly smaller than that of crystalline AChE@CMOF‐74 (4.7 µm) (Figure 1b,d,f). Energy‐dispersive spectroscopy (EDS) elemental mapping of AChE@AMOF‐74 and AChE@CMOF‐74 confirmed the distribution of Zn and N, originating from both MOF and protein components (Figure S6, Supporting Information). Fourier‐transform infrared (FT‐IR) spectra of both AChE@CMOF‐74 and AChE@AMOF‐74 exhibited characteristic amide I and amide II bands at 1655 and 1547 cm^−1^, respectively, corresponding to the enzyme backbone and providing further evidence for the successful incorporation of AChE (Figure S7, Supporting Information). X‐ray photoelectron spectroscopy (XPS) further revealed the presence of characteristic peaks of Zn 2p and N 1s, confirming the successful incorporation of AChE into AMOF‐74 (Figure S8, Supporting Information). To evaluate the accessibility of AChE within AChE@AMOF‐74, Rhodamine B (RhB)‐labeled AChE was selected to trace the distribution using confocal microscopy. The fluorescence images revealed a homogeneous distribution of AChE within the MOFs (Figure 1g). Based on high‐resolution transmission electron microscopy (TEM) images, AChE@AMOF‐74 was found to exhibit obvious amorphous properties, characterized by the absence of lattice fringes, contrasting sharply with the clear 0.28 nm regular lattice crystal structure of AChE@CMOF‐74 (Figure 1c,e). Additionally, X‐ray diffraction (XRD) patterns of AChE@AMOF‐74 suggested the nanosized particles with amorphous structure (no distinct 2θ peaks), while AChE@CMOF‐74 holds typical 2θ peaks at 38.4° and 44.7° that confirm the high crystallinity (Figure 1h).

The formation of enzyme@MOFs is primarily driven by the interactions among metal nodes, enzymes, and organic linkers.^[^ ^33^ ^]^ Different from loading‐type frameworks for enzyme encapsulation (such as ZIF‐8 and ZIF‐90),^[^ ^34^, ^35^ ^]^ Zn^2+^ as a node between AChE and H_4_DOBDC could disrupt the initial coordination configuration and reduce the coordination saturation of Zn‐H_4_DOBDC (Figure S9, Supporting Information), effectively regulating the crystallization process. High‐resolution XPS spectra showed that the characteristic peak of Zn 2p_3/2_ gradually shifted from 1021.5 eV to a higher energy of 1025.3 eV as the AChE@AMOF‐74 transitions to AChE@CMOF‐74 (Figure 1i). This indicated the formation of new Zn─O coordination bonds through competitive coordination of Zn^2+^ with ─C─OH and ─C═O groups shared between H4DOBDC and AChE. Similarly, the Zn─N binding energy of N 1s gradually shifted from 401.6 eV to a higher energy of 402.2 eV, which further demonstrated the existence of a coordination relationship between Zn and AChE (Figure S10, Supporting Information). Additionally, FT‐IR results showed the absorption peaks at 1385 and 1356 cm^−1^, corresponding to ─CH_3_ stretching vibration and aromatic compound symmetric stretching vibration, had disappeared in AChE@AMOF‐74 (Figure 1j). Extended X‐ray absorption fine structure (EXAFS) analyses were conducted to probe the Zn coordination environment. In the k^2^‐weighted FT‐EXAFS spectra (Figure S11, Supporting Information), both AChE@AMOF‐74 and AChE@CMOF‐74 exhibit a Zn─N/O scattering peak at ∼1.56 Å, indicating a similar average Zn─N/O bond length. EXAFS fitting reveals that the Zn─N/O coordination number in AChE@AMOF‐74 is 4.0, lower than the 4.4 observed in AChE@CMOF‐74, suggesting the presence of under‐coordinated Zn sites in the amorphous framework. The weakening or even disappearance of the Zn─Zn scattering in AChE@AMOF‐74 further confirms the loss of long‐range order in the amorphous framework (Figure 1k; Table S1, Supporting Information). Consistently, wavelet transform (WT) analysis further reveals that the maximum intensity of the Zn‐N/O scattering in k‐space is higher for AChE@CMOF‐74, reflecting an more ordered local coordination environment in the crystalline MOF (Figure 1l; Figure S12, Supporting Information). NH_3_ temperature‐programmed desorption (NH_3_‐TPD) results reveal distinct desorption behaviors between the two materials. In the low‐temperature region (∼118.7 °C, corresponding to COOH‐NH_3_ sites), AChE@AMOF‐74 displays a total NH_3_ desorption of 621 µmol g^−1^, significantly higher than 210 µmol g^−1^ for AChE@CMOF‐74, indicating a greater abundance of uncoordinated ─COOH groups in the amorphous framework (Figure 1m). In the high‐temperature region (∼296.6 °C, corresponding to Zn^2+^‐NH_3_ sites), AChE@CMOF‐74 exhibits a total NH_3_ desorption of 659 µmol g^−1^, whereas AChE@AMOF‐74 shows only a weak peak. These results indicate that the high Zn^2+^ concentration in AChE@CMOF‐74 facilitates stable Zn‐H_4_DOBDC coordination, thereby promoting the formation of a dense crystalline framework. In contrast, defect‐engineered strategy encourages the competitive coordination of Zn^2+^ with both AChE and H_4_DOBDC, thereby introducing defects into the crystalline MOF framework and imparting additional amorphous characteristics. Previously reported AMOFs were commonly synthesized through post‐synthetic treatments such as high pressure,^[^ ^36^, ^37^ ^]^ ball milling,^[^ ^38^ ^]^ or heating,^[^ ^37^ ^]^ which are generally unfavorable for enzyme immobilization. Such a AChE@AMOF‐74 was directly synthesized under mild aqueous conditions by a defect‐engineered strategy, providing an enzyme‐friendly and operationally convenient protocol.

### 2.2 Morphological Evolution of Defect‐Engineered AChE@AMOF‐74

**Figure 2:**
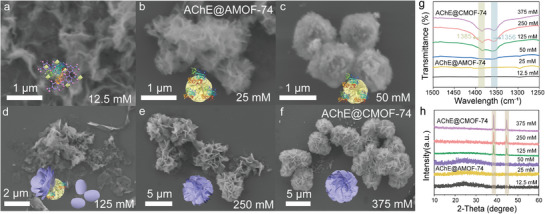
Defect engineering of AChE@MOF‐74 by regulating the concentration of Zn^2+^. a–f) The SEM images reveal the morphological evolution of AChE@MOF‐74 at different Zn^2+^ concentrations (12.5‐375 mm). The sample obtained at 25 mm Zn^2+^ is designated as AChE@AMOF‐74, whereas that prepared at 375 mm Zn^2+^ is designated as AChE@CMOF‐74. g) FT‐IR spectra of AChE@MOF‐74 at different Zn^2+^ concentration. h) XRD spectrum of AChE@MOF‐74 at different Zn^2+^ concentration.

To elucidate the effect of Zn^2+^ concentration on the formation mechanism of structural defects, we systematically investigated the nanostructural evolution of AChE@MOF‐74 composites under different Zn^2+^ (12.5–375 mm) concentrations by regulating the kinetic parameters of the co‐assembly process (**Figure** **2**a–f). As shown in Figure 2a, under low Zn^2+^ concentrations (∼12.5 mm), amorphous aggregates with irregular morphologies were observed, which were most likely ternary complexes of Zn^2+^, H_4_DOBDC, and AChE that may spontaneously form phase separation in a solution (Figure S13, Supporting Information). As the Zn^2+^ concentration increased to 25 mm, the nanocomposites gradually evolved into a loosely organized, porous AChE@AMOF‐74 (Figure 2b). Upon further increasing the Zn^2+^ concentration to 50 mm, the porous AChE@AMOF‐74 gradually transformed into a dense morphology with an average particle size of 1.2 µm (Figure 2c; Figure S14, Supporting Information). At this stage, the primary role of increasing Zn^2+^ concentration was to promote the growth of AChE@AMOF‐74. When the Zn^2^⁺ concentration reached 125 mm, aggregation of the Zn^2^⁺‐H_4_DOBDC complexes led to the formation of nanopetals (Figure 2d,g), which inserted into the amorphous phase of the nanocomposite to form a hierarchical structure. Remarkably, crystalline material was observed in the petal‐like structures, indicating that crystallinity was predominantly localized within the petals (Figure 2h). EDS elemental mapping reveals that little N elements (ascribed to AChE) were present in the nanopetals (Figure S15, Supporting Information). That is, the enzyme is not involved in the formation of nanopetals. The nanopetals have multiplied as the Zn^2+^ concentration increased to 375 mm, gradually densifying the nanoflower‐like structure of the nanocomposite by utilizing enzymes as “glue” to bind them together, ultimately forming a multi‐layer structure with AChE@AMOF‐74 as the core and nanopetals as the shell (Figure 2e,f). Based on these observations, we propose that the mechanism by which Zn^2+^ concentration influences the degree of structural defects can be divided into two distinct stages: i) At relatively low Zn^2+^ concentrations, the competitive effect of AChE reduces the coordination saturation between Zn^2+^ and H_4_DOBDC, leading to the formation of a porous flower‐core structure in AChE@AMOF‐74. ii) As the Zn^2+^ concentrations increase, crystalline nanoflakes initially form and subsequently embed into the AChE@AMOF‐74 framework, giving rise to the hierarchical nanoflower structure characteristic of AChE@CMOF‐74.

### 2.3 High Activity of AChE@AMOF‐74

**Figure 3:**
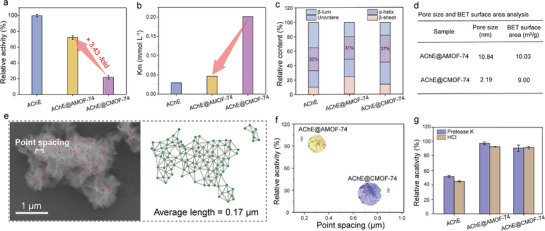
Structural and functional evaluation of AChE@AMOF‐74. a) Relative activity of AChE, AChE@AMOF‐74 and AChE@CMOF‐74 (the activity of free AChE is defined as 100%). b) K_m_ of AChE, AChE@AMOF‐74 and AChE@CMOF‐74. c) Secondary structure compositions of AChE, AChE@AMOF‐74 and AChE@CMOF‐74. b) Pore size and BET surface area analysis of AChE@AMOF‐74 and AChE@CMOF‐74. e) Results of Delaunay triangulation applied to AChE@AMOF‐74. f) Relative activity of AChE@AMOF‐74 and AChE@CMOF‐74 with varying point spacing. g) Stability of AChE, AChE@AMOF‐74 and AChE@CMOF‐74 after incubation with proteinase K or HCl for 30 min.

The coordination defects in the framework, which facilitate enzyme loading and substrate transport, prompted us to evaluate the enzymatic activity of the AChE@AMOF‐74 composites. Surprisingly, AChE@AMOF‐74 retained 72% relative activity of the free enzyme, which was 3.4‐fold increase compared with AChE@CMOF‐74 and higher activity than other nanocomposites (**Figure** **3**a; Figure S16, Supporting Information). Consistently, AChE@AMOF‐74 exhibited an enzyme loading efficiency of 82%, markedly higher than that of AChE@CMOF‐74 (Figure S17, Supporting Information). Further enzymatic kinetic studies revealed that the Michaelis‐Menten constant (K_m_) of AChE@AMOF‐74 (0.04645 mmol L^−1^) closely resembled that of free AChE (0.02955 mmol L^−1^), indicating a comparable substrate affinity of free AChE and AChE@AMOF‐74 composites (Figure 3b; Figure S18, Supporting Information). In contrast, for AChE encapsulated in CMOF‐74, K_m_ was increased to 0.2015 mmol L^−1^, suggesting a severe limitation for substrate transportation. Second‐derivative FT‐IR spectra showed a strong amide II absorption at 1559.2 cm^−1^ for free AChE. The peak position of AChE@AMOF‐74 remained nearly unchanged, while that of AChE@CMOF‐74 exhibited a slight red shift to 1558.7 cm^−1^ (Figure S19, Supporting Information). The change in secondary structure content of the composite was analyzed through Gaussian multi‐component fitting. The α‐helix structure of AChE@AMOF‐74 closely resembles that of free AChE, thereby largely preserving its enzymatic activity (Figure 3c; Figure S20, Supporting Information). These results indicated that the suitable microenvironment of AMOF‐74 had little influence on the structure of AChE and provided high substrate transport activity. To elucidate the structural basis of the enhanced activity, the pore characteristics induced by defect engineering were analyzed. Nitrogen adsorption analysis and BJH pore size distribution revealed that AChE@AMOF‐74 possessed mesopores with an average diameter of ≈10.8 nm, which is markedly larger than that of AChE@CMOF‐74 (2.2 nm), and also exhibited a slightly higher specific surface area (Figure 3d). Additionally, statistical analysis was conducted to examine the porosity of AChE@MOF‐74 under different growth conditions. Specifically, the “point spacing” between flower‐leaf junctions with distinct surface features was measured as a structural parameter (Figure 3e), where shorter spacing corresponds to higher porosity.^[^ ^39^ ^]^ Distances between adjacent points were calculated by applying Delaunay triangulation,^[^ ^40^ ^]^ in which three points form a triangle without any other point inside its circumcircle, thereby yielding non‐overlapping triangular meshes (Figure S21, Supporting Information). The edge lengths of these triangles were then calculated and used as the inter‐point distances. Using this protocol, the point spacing of individual AChE@AMOF‐74 particles was obtained (Figure S21, Supporting Information). However, when analyzing point spacing across numerous nanoparticles, Delaunay triangulation may generate erroneous long‐distance connections between discrete particles. To correct for such artifacts and improve measurement accuracy, a threshold based on the upper bound of the 95% confidence interval of a normal distribution was introduced (Figure 3e; Figure S22, Supporting Information). The average point spacing revealed that AChE@AMOF‐74 exhibited the shortest point spacing and the highest enzyme activity (Figure 3f). The reduced point spacing indicates a higher porosity, resulting in a more porous and efficient MOF structure that enhances the catalytic performance of the enzyme. ^[^ ^39^ ^]^ These results suggest that the enhanced biocatalytic performance of AChE@AMOF‐74 arises from three interrelated effects induced by coordination defects: i) the amorphous structure of AMOF‐74 shortens substrate diffusion pathways, facilitating rapid enzyme‐substrate interactions; ii) the formation of mesopores provides a uniform and accessible spatial environment for AChE, thereby improving enzyme loading efficiency; and iii) the flexible amorphous framework imposes minimal structural constraints on the tertiary conformation of AChE, effectively preserving its catalytic activity.

Furthermore, an ideal enzyme immobilization strategy aims to maintain the catalytic activity while optimizing its stability. After exposure to proteinase K (0.1 mg mL^−1^) and hydrochloric acid (0.01 m), AChE@AMOF‐74 exhibited markedly improved stability relative to free AChE (Figure 3g). Interestingly, no significant differences in protection were observed between AMOF‐74 and the other structures, indicating that different structures of MOF‐74 provide effective and comparable protection against external macromolecular proteinases and acidic conditions (Figure S23, Supporting Information).

### 2.4 Construction of the AChE@AMOF‐74‐Based Biosensor

**Figure 4:**
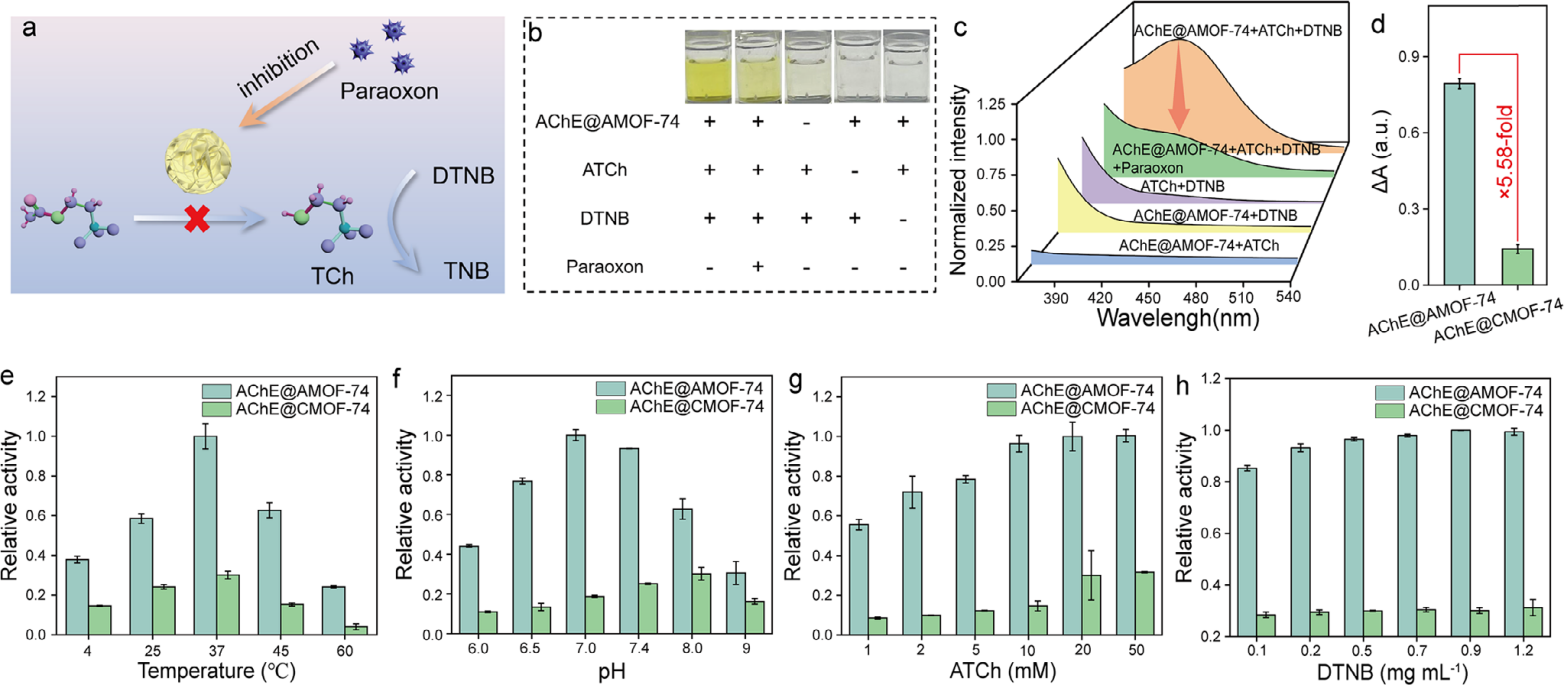
Construction of the AChE@AMOF‐74‐based biosensor. a) Schematic illustration of the colorimetric sensing platform based on the AChE@AMOF‐74 composite for pesticide detection. b) Feasibility verification of the AChE@AMOF‐74‐based biosensor through a colorimetric response test. c) Ultraviolet spectrum of AChE@AMOF‐74‐based biosensor with the paraoxon. d) Absorbance changes in the reaction systems of AChE@AMOF‐74 and AChE@CMOF‐74 under 50 ng mL^−1^ paraoxon inhibition conditions. e) The relative activity of AChE@AMOF‐74 and AChE@CMOF‐74 at different reaction temperatures. f) The effect of incubation pH on the catalytic activity of AChE@AMOF‐74 and AChE@CMOF‐74. g) The effect of DTNB concentration on the activity of AChE@AMOF‐74 and AChE@CMOF‐74. h) The effect of ATCh concentration on the activity of AChE@AMOF‐74 and AChE@CMOF‐74.

To elucidate the influence of composite architecture on the performance of pesticide detection, paraoxon was selected as a model analyte to evaluate the sensing capabilities of an AChE@MOF‐74‐based biosensor. The sensing mechanism is based on the AChE‐catalyzed hydrolysis of acetylthiocholine (ATCh) to generate thiocholine (TCh), which reacted with 5,5′‐dithiobis (2‐nitrobenzoic acid) (DTNB) to produce a colorimetric signal (Figure S24, Supporting Information). Paraoxon inhibited the AChE activity, thereby reducing the signal intensity (**Figure** **4**a). The feasibility of the sensing mechanism was confirmed through color changes (Figure 4b) and UV–vis absorption spectra (Figure 4c). As shown in Figure 4b, a distinct yellow color was observed when AChE@AMOF‐74, ATCh, and DTNB were simultaneously present, indicating the formation of 2‐nitro‐5‐thiobenzoate (TNB). The addition of paraoxon resulted in a decrease in yellow color intensity, indicating inhibition of AChE activity (Figure 4c). Subsequently, the pesticide response of AChE@AMOF‐74 and AChE@CMOF‐74 was evaluated. Interestingly, AChE@AMOF‐74 with an amorphous structure exhibited a 5.6‐fold higher response to paraoxon in comparison to AChE@CMOF‐74 (Figure 4d).

The colorimetric response of the biosensor is influenced by working parameters, e.g., pH, temperature, and substrate concentration. Optimization of these parameters can enhance biosensor performance. The catalytic activity of AChE@AMOF‐74 and AChE@CMOF‐74 was systematically evaluated under different reaction conditions to provide a comprehensive comparison. As shown in Figure 4e, the catalytic activity of AChE@AMOF‐74 increased with temperature and exhibited the highest activity at 37 °C. The effect of pH was examined over the range of 6.0 to 9.5, with the AChE@AMOF‐74 exhibiting maximum enzymatic activity at pH 7.0 (Figure 4f). Furthermore, the relative activity of AChE@AMOF‐74 increased with increasing DTNB concentration from 0 to 0.9 mg mL^−1^. However, when the concentration exceeded 0.9 mg mL^−1^, the activity remained almost constant, indicating that the substrate concentration had reached saturation (Figure 4g). Similarly, the relative activity stabilized when the ATCh concentration reached 10 mm, suggesting that this concentration was sufficient to fully activate AChE (Figure 4h). Notably, owing to the structural advantages of the amorphous framework, AChE@AMOF‐74 exhibits 1.88‐6.57‐fold higher catalytic activity than AChE@CMOF‐74 across all tested conditions

**Figure 5:**
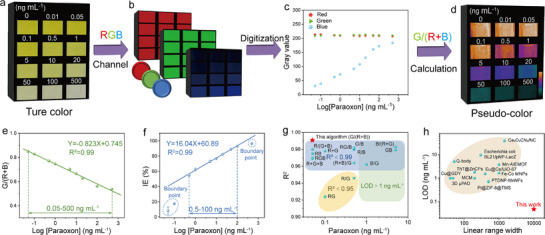
Sensing performance of the AChE@AMOF‐74‐based biosensor. a) True‐color images of AChE@AMOF‐74 system under varying paraoxon concentrations (0–500 ng mL^−1^). b) RGB channel images of the AChE@AMOF‐74 system under varying paraoxon concentrations. c) The dependence of paraoxon concentration on the R, G, and B values. d) The calculation of the G/(R + B) ratio to construct pseudo color representations. e) The relationship of the G/(R + B) response with paraoxon concentration. f) The linear relationship between inhibition efficiency (IE (%)) and paraoxon concentration. g) Comparison of detection limits and linearity between the G/(R + B) algorithm and other RGB image processing algorithms. h) Comparison of limit of detection (LOD) and linear range width with previously reported biosensors.

Leveraging the high sensitivity and stability of the AChE@AMOF‐74 composite, an enzyme‐based biosensor was developed for on‐site detection of paraoxon. Under optimal reaction conditions, the absorbance at 412 nm progressively decreased with increasing paraoxon concentrations (0–500 ng mL^−1^), indicating a concentration‐dependent inhibition of AChE activity within the AChE@AMOF‐74 composite (Figure S25, Supporting Information). To enable rapid signal readout for POC applications, the colorimetric response was converted into a distinct color transition signal. Maintaining a fixed configuration of the light source, sample, and smartphone, true‐color images were consistently captured in the presence of the analyte (**Figure** **5**a). An image digitization algorithm was applied to extract accurate data. The color signal was decomposed into its three primary channels (RGB: red, green, and blue) using ImageJ software, and the corresponding RGB values were obtained (Figure 5b). Upon exposure to paraoxon, the color responses in each digital channel exhibited concentration‐dependent variations (Figure 5c). To better visualize these findings, the color responses of the samples were further digitized and processed into digital color difference maps (pseudo‐color images) (Figure 5d). Various algorithms (RGB combinations, grayscale, and HSV‐based processing models) were quantitatively analyzed for their linear relationship with paraoxon concentration through fitting procedures (Figure 5g; Figure S26, Supporting Information). The fitting results indicate that the relationship between G/(R+B) and paraoxon concentration follows the linear equation Y = 0.823 Log[paraoxon] + 0.745 (Figure 5e) with a high correlation coefficient (R^2^ = 0.99). Notably, the linear detection range was 0.05–500 ng mL^−1^, which is 50‐fold broader than that of the conventional colorimetric sensing system (0.5–100 ng mL^−1^) (Figure 5f). The detection limit was as low as 0.05 ng mL^−1^, outperforming other color processing algorithms (Figure 5g; Figure S26 and Table S2, Supporting Information), which is tenfold lower than that of the conventional inhibition efficiency (IE (%))‐based algorithms using Equation S1 (Supporting Information) (Experimental section). Compared with previously reported pesticide sensors, this sensing platform demonstrates significant improvements in sensitivity and linear range width (defined as the ratio of the upper detection limit to the lower detection limit), enabling quantitative detection of paraoxon (Figure 5h; Table S3, Supporting Information). The enhanced performance can be attributed to three key factors: i) the AChE@AMOF‐74 exhibits excellent pesticide recognition capability, which benefits its application in biosensing; ii) the AChE@AMOF‐74 high catalytic activity and superior stability enable long‐term reliable operation and improved signal amplification; iii) the integration with image processing algorithms allows precise quantification and computational correction of subtle colorimetric changes, enhancing sensitivity, broadening the linear range, and improving the reliability of quantitative detection.

### 2.5 Practical Performance of Biosensor

**Figure 6:**
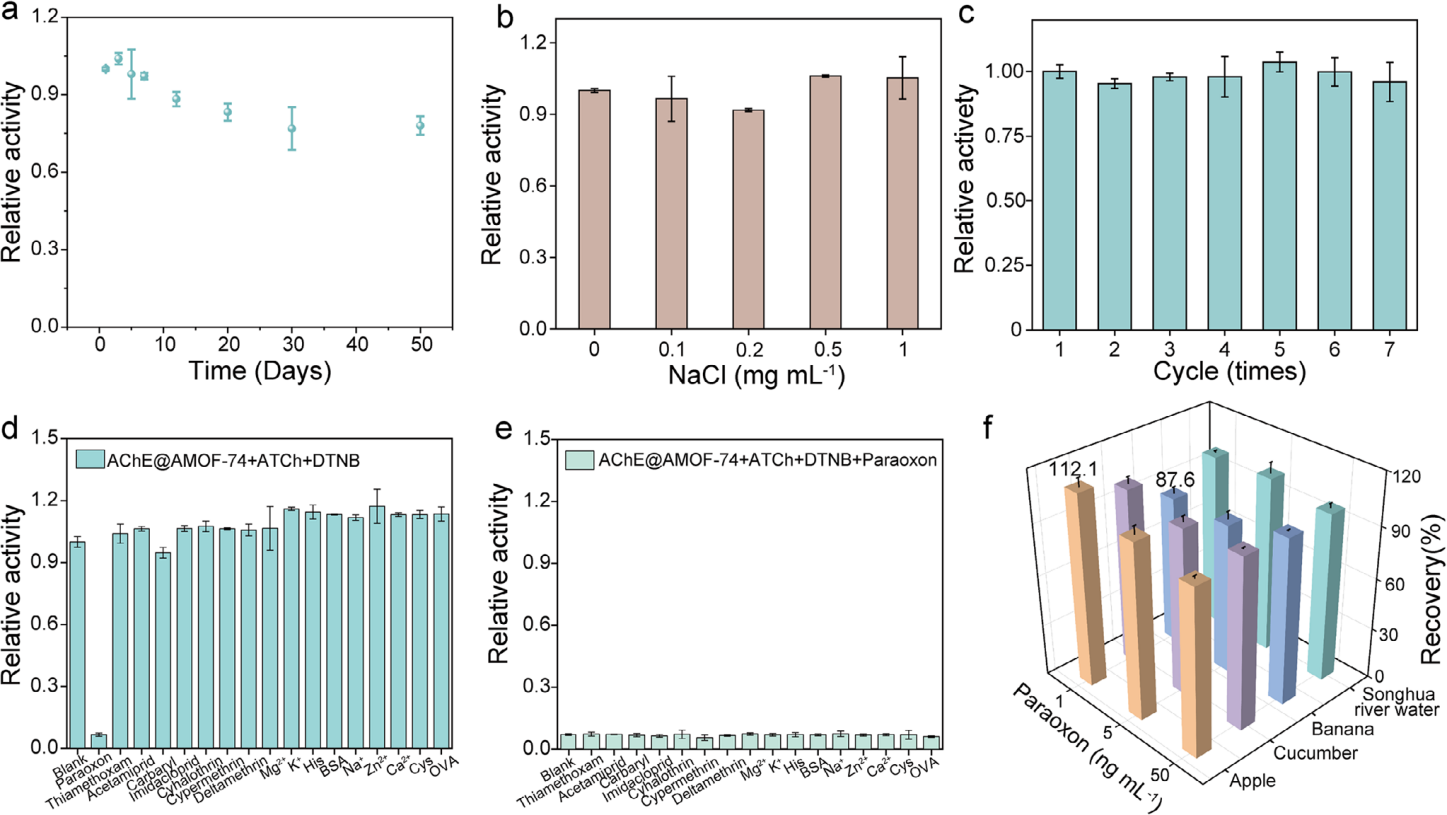
Practical applications of AChE@AMOF‐74‐based sensing platform. a) The long‐term operational stability of AChE@AMOF‐74. b) Stability of AChE@AMOF‐74 under different NaCl concentrations. c) Recyclability of AChE@AMOF‐74. d) Selectivity of AChE@AMOF‐74 for OPs detection (paraoxon concentration is 100 ng mL^−1^, other pesticide concentrations are 1000 ng mL^−1^). e) Anti‐interference ability of AChE@AMOF‐74. f) Determination of paraoxon in spiked samples.

The long‐term operational stability of AChE@AMOF‐74‐based biosensor is a critical factor for its practical usability and reliability of biosensors. Therefore, the activity retention of the biosensor was investigated over different storage periods. Interestingly, the AChE@AMOF‐74 biosensor retained more than 80% of its initial activity after 50 days of storage (**Figure** **6**a), demonstrating superior stability compared to previously reported enzyme‐based biosensors. ^[^ ^41^, ^42^ ^]^ In practical applications, target samples such as food and plants are often exposed to complex environments, including high salinity or alkaline conditions. Therefore, achieving accurate pesticide detection under these conditions is crucial for ensuring the reliable performance and broad applicability of the biosensor. The effect of NaCl concentrations (0–1 mg mL^−1^) on biosensor was investigated. As shown in Figure 6b, AChE@AMOF‐74 retains good stability even at high NaCl concentrations, demonstrating its high tolerance to salinity. Recyclability is a key parameter for evaluating the performance of MOF‐immobilized enzymes. ^[^ ^43^ ^]^ AChE@AMOF‐74 maintained over 90% catalytic activity even after seven cycles of operation, demonstrating excellent reusability and operational stability (Figure 6c). The exceptional stability of AChE@AMOF‐74 can be attributed to the robust AMOF‐74 framework (Figure S27, Supporting Information), which provides effective protection for the encapsulated enzyme. These findings suggest that the AChE@AMOF‐74‐based biosensor is effective in maintaining the integrity of the AChE structure, thus providing long‐term stability.

Selectivity and anti‐interference ability are crucial parameters in evaluating the sensor performance in the analysis of complex sample. To simulate the environmental conditions, a variety of potential interferents were tested, including common pesticides (e.g., thiamethoxam, acetamiprid, carbaryl, imidacloprid, cyhalothrin, cypermethrin, and deltamethrin) and substances in food and environmental matrices (e.g., metal ions, proteins, amino acids, and vitamins). As shown in Figure 6d, despite being present at a tenfold higher concentration (1000 ng mL^−1^) than paraoxon (100 ng mL^−1^), the response signals from these interferents were comparable to that of the blank. In terms of anti‐interference ability, the signal of the AChE@AMOF‐74 system remained unaffected by the presence of the aforementioned substances during the detection process, demonstrating its robust resistance to interference (Figure 6e). Such remarkable anti‐interference ability may be attributed to the specific recognition ability of AChE and the protective effect of the AMOF‐74 structure. To demonstrate the biosensor's potential applicaiton, it was applied to detect paraoxon in various food and environmental samples, including apple, cucumber, banana, and Songhua River water. The standard solutions of paraoxon (1.0, 5.0, and 50 ng mL^−1^) are added to these samples. The recoveries of paraoxon in these real samples ranged from 87.6% to 112.1%, with relative standard deviations (*n* = 3) below 6.20%, indicating that the AChE@AMOF‐74 sensor platform exhibits satisfactory accuracy in complex matrices (Figure 6f).

## 3 Conclusion

In summary, we developed an AChE@AMOF‐74‐based biosensor with excellent pesticide recognition capability for the on‐site detection of OPs. The AChE@AMOF‐74 structure was fabricated by a defect‐engineered strategy to provide a suitable microenvironment and obtain high porosity for enzyme immobilization. Impressively, AChE@AMOF‐74 demonstrates markedly enhanced performance, exhibiting 3.4‐fold higher enzymatic activity and a 5.6‐fold greater recognition capability toward paraoxon compared with AChE@CMOF‐74. Such a superior sensing performance of AChE@AMOF‐74 is attributed to its amorphous structure, which increases pore size and porosity, facilitating substrate recognition and amplifying recognition signals. To improve the practical applicability of the biosensor and achieve accurate on‐site quantitative analysis, a digital image processing algorithm was employed to convert colorimetric image parameters into quantifiable numerical data. This digitization approach enables precise quantification and computational correction of colorimetric changes, achieving a LOD as low as 0.05 ng mL^−1^, which is tenfold lower than conventional colorimetric systems. Notably, the biosensor achieves a wide detection range from 0.05 to 500 ng mL^−1^, 50‐fold broader than conventional systems, thereby enhancing its applicability in environments with variable pesticide levels. Benefiting from these structural characteristics, the developed AChE@AMOF‐74‐based biosensor exhibits good long‐term stability and high tolerance to environments. This study not only provides a convenient in situ immobilization strategy for constructing enzyme@AMOF composites with both stability and sensitivity, but also demonstrates significant potential for on‐site biosensor applications. We anticipate that integrating the AChE@AMOF‐74‐based sensor with image processing algorithms will greatly advance environmental monitoring and precision agriculture.

## 4 Experimental Section

### Preparation of AChE@AMOF‐74

The AChE@AMOF‐74 was prepared by dissolving Zn(NO_3_)_2_·6H_2_O (25 mm), AChE (1 mg mL^−1^), and H_4_DOBDC (25 mm) in 8.0 mL of 50 mm Tris‐HCl buffer (pH 8.0) under stirring for 10 min. The resulting mixture was centrifuged at 4320 rpm for 10 min, and the precipitate was washed and redispersed in 2 mL ultrapure water. During the self‐assembly of AChE@AMOF‐74, H_4_DOBDC served not only as the organic linker but also as a coordination modulator. The carboxyl and phenolic hydroxyl groups of H_4_DOBDC competed with amino and carboxyl residues on the AChE surface for Zn^2^⁺ coordination sites. This competitive coordination perturbed the regular Zn─O bonding network of the MOF framework, thereby inducing coordination defects and enabling the in situ encapsulation of AChE within the amorphous structure.

### Preparation of AChE@CMOF‐74

The AChE@CMOF‐74 was prepared by dissolving Zn(NO_3_)_2_·6H_2_O (375 mm), AChE (1 mg mL^−1^), and H_4_DOBDC (25 mm) in 8.0 mL of 50 mm Tris‐HCl buffer (pH 8.0) under stirring for 10 min. The resulting mixture was centrifuged at 4320 rpm for 10 min, and the precipitate was washed and redispersed in 2 mL ultrapure water.

### Enzyme Activity Evaluation

Twenty‐five microliters of different composites (AChE, AChE@AMOF‐74, and AChE@CMOF‐74) were mixed with 50 µL of 10 mm PBS (pH = 7.0), 50 µL of ultrapure water, 50 µL of ATCh (20 mm), and 50 µL of DTNB (0.9 mg mL^−1^). Subsequently, the mixture was incubated in an incubator at 37 °C for 30 min and the absorbance was recorded at 412 nm.

### Stability Evaluation

To assess enzymatic stability, 25 µL of different composites (AChE, AChE@AMOF‐74, and AChE@CMOF‐74) was separately treated with 25 µL of proteinase k or HCl for 30 min. Subsequently, 50 µL of PBS (10 mm), 50 µL of ATCh (10 mm), 50 µL of water, and 50 µL of DTNB (0.9 mg mL^−1^) were added and mixed homogeneously at 37 °C for 30 min. The absorbance was measured at 412 nm.

### Detection of Paraoxon

For pesticide detection, 25 µL of AChE@AMOF‐74 was incubated with 25 µL of paraoxon standard solution for 40 min. After incubation, 50 µL of 10 mm PBS (pH 7.0), 25 µL of ultrapure water, 50 µL of ATCh (10 mm), and 50 µL of DTNB (0.9 mg mL^−1^) were added. The mixture was incubated at 37 °C for 30 min, and the absorbance was measured at 412 nm. The inhibition efficiency (IE%) was calculated to quantify the pesticide concentration, using the following equation:

(1) $$IE\left(\%\right)=\frac{\left(A-A_{1}\right)}{\left(A-A_{0}\right)}\times 100\%$$

where A_0_ is the baseline absorbance of the sensing system (blank), A is the absorbance without paraoxon, and A_1_ is the absorbance after exposure to different concentrations of paraoxon.

## Conflict of Interest

The authors declare no conflict of interest.

## Supporting information



Supporting Information

## Data Availability

The data that support the findings of this study are available from the corresponding author upon reasonable request.
