# Supplementary material for: Enhanced Recognition via Defect Engineered Amorphous Metal–Organic Framework for Sensitive and Stable Pesticide Biosensor
Source: Adv Sci (Weinh). 2025 Nov 18;13(7):e17919. doi: 10.1002/advs.202517919 (PMC12866820; doi:10.1002/advs.202517919)
Supplement: Supplementary file 1 — Supporting Information [file ADVS-13-e17919-s001.docx]

**Supporting Information**

**Enhanced Recognition via Defect Engineered Amorphous Metal–Organic Framework for Sensitive and Stable Pesticide Biosensor**

Changshun Su^1^, Xiangyu Zhai^1^, Meng Zhang^1^, Mengxue Li^2^, Xinru Zhang^3^, Chunyan Sun^1^, Xu Yan^2,^*, Hongxia Li^1,^*

^1^ Department of Food Quality and Safety, College of Food Science and Engineering, Jilin University, Changchun 130062, China

^2^ State Key Laboratory on Integrated Optoelectronics, Key Laboratory of Advanced Gas Sensors of Jilin Province, College of Electronic Science & Engineering, Jilin University, Changchun 130012, P. R. China

^3^ Department of Pharmacy, the second hospital of Jilin University

* Corresponding author

Email: hxiali@jlu.edu.cn

yanx@jlu.edu.cn

**Chemicals and materials**

Zinc nitrate [Zn(NO_3_)_2_•6H_2_O] was purchased from Aladdin Chemical Reagent Co., Ltd. (Shanghai, China). 2,5-Dihydroxyterephthalic acid (H_4_DOBDC), acetylthiocholine (ATCh), acetylcholinesterase (AChE), Tris-HCl, and 5, 5'-Dithiobis-(2-nitrobenzoic acid) (DTNB) were all purchased from Shanghai Macklin Biochemical Technology Co., Ltd. Bovine serum albumin (BSA) were obtained from Ryon Biological Technology Co. Ltd. (Shanghai, China). CaCl_2_ were got from Aladdin Reagent Co. Ltd (Shanghai, China). Cysteine (Cys), Glucose (Glu), Whey protein, Ovalbumin (OVA), Trypsin (Try), and proteinase K were obtained from Beijing Dingguo Changsheng Biotechnology Co., Ltd. Paraoxon, isoprocard, deltamethin, imidacloprid, acetamiprid, thiamethoxam, cyhalothrinnd and other pesticide samples were obtained from Tianjin Zhongyi technology Co., Ltd.

**Apparatus**

The microstructures are recorded using JEM-2100 transmission electron microscope (TEM) and JEM-7500 scanning electron microscope (SEM). X-ray photoelectron spectroscopy (XPS) measurement is performed using an American Thermo Fisher 250XI photoelectron spectrometer. The phase and crystalline information of the samples is examined by XRD (Rigaku D/MAX-2550, Cu-Kα, λ = 1.5418 Å). Fourier Transform Infrared (FT-IR) spectra were recorded on a Shimadzu IRPrestige-21 spectrometer. The absorption spectrum was measured by Shimadzu UV-270001 ultraviolet-visible spectrophotometer. The absorbance values were recorded on a Thermo Fisher (Shanghai) multifunctional microplate reader Varioskan LUX.

**Enzyme kinetic studies**

The kinetic behavior of AChE@MOF-74 immobilized acetylcholine ester was investigated and the performance of immobilized acetylcholine ester was evaluated. 25 μL of catalyst, containing AChE@AMOF-74, AChE@TMOF-74, AChE@ZIF-8, ZIF-8, and AChE, respectively, were added to a mixture containing 50 μL of PBS (10 mM, pH = 7.0), 50 μL of water, 50 μL of ATCh at different concentrations, and 50 μL of DTNB (0.9 mg mL^-1^) in a mixed solution. The change in absorbance with time was immediately detected and the kinetic curve was plotted. The kinetic parameters (K_m_ and V_max_) are calculated by the Michaelis-Menten equation:

1/V = (K_m_ / V_max_) (1/[S] + 1/V_max_)

where [S] stands for the concentration of substrate, V stands for the reaction rate at this concentration of the substrate.

**The sensing mechanism**

In this work, acetylcholinesterase (AChE) could catalyze hydrolysis acetylthiocholine to produce TCh, which regulates the absorbance intensity. The reaction that is catalyzed by AChE i:


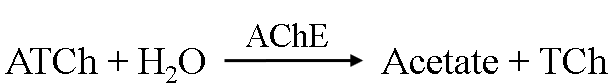


Specifically, AChE catalysis occurs when the anionic binding site of the catalytic coordinated triad (histidine, serine, and aspartic acid) attracts the positively charged thiocholine. The serine hydroxyl group attacks and cleaves the ester after its deprotonation by a neighboring histidine group in the triad. Thus, the TCh could trigger the decomposition of DTNB to TNB with a characteristic absorption peak around 412 nm, accompanying a distinguishable color change.


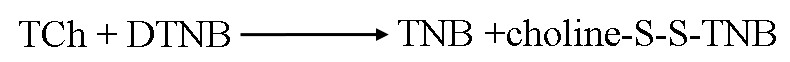


Paraoxon could covalently bind the triad's anionic binding site of AChE to form a phosphorylated enzyme, blocking the activity of the enzyme and inducing the change of absorbance intensity of the system.

**Recovery rate calculation**

The recovery rate refers to the percentage of an analyte that is accurately measured or detected by a sensor after being added to a sample. The recovery rate is calculated using the following formula:

$$Recovery rate=\frac{C_{m}}{C_{a}}\times100\%$$

*C_m_* is the actual measured concentration; *C_a_* is the concentration of pesticide added to the sample.

**Real sample detection**

To evaluate the practical applicability of the biosensor, real samples, including apple, cucumber, banana, and Songhua River water were tested. Songhua River water was filtered through a 0.22 μm membrane to remove particulates and then spiked with paraoxon at concentrations of 1.0, 5.0, and 50.0 ng mL^-1^. For fruit and vegetable samples, pretreatment was performed according to the Chinese National Standard GB/T 5009.199-2003 (“Rapid Detection of Organophosphorus and Carbamate Pesticide Residues in Vegetables”). A phosphate buffer (pH 8.0) was prepared by dissolving 11.9 g of anhydrous dibasic potassium phosphate and 3.2 g of monobasic potassium phosphate in 1000 mL of distilled water. Representative samples were washed to remove surface contaminants and cut into ~1 cm^2^ pieces. After spiking with paraoxon at the same concentrations (1.0, 5.0, and 50.0 ng mL^-1^), 5 mL of the buffer was added. The mixtures were shaken for 10 min, and the resulting extracts were collected for analysis.

**
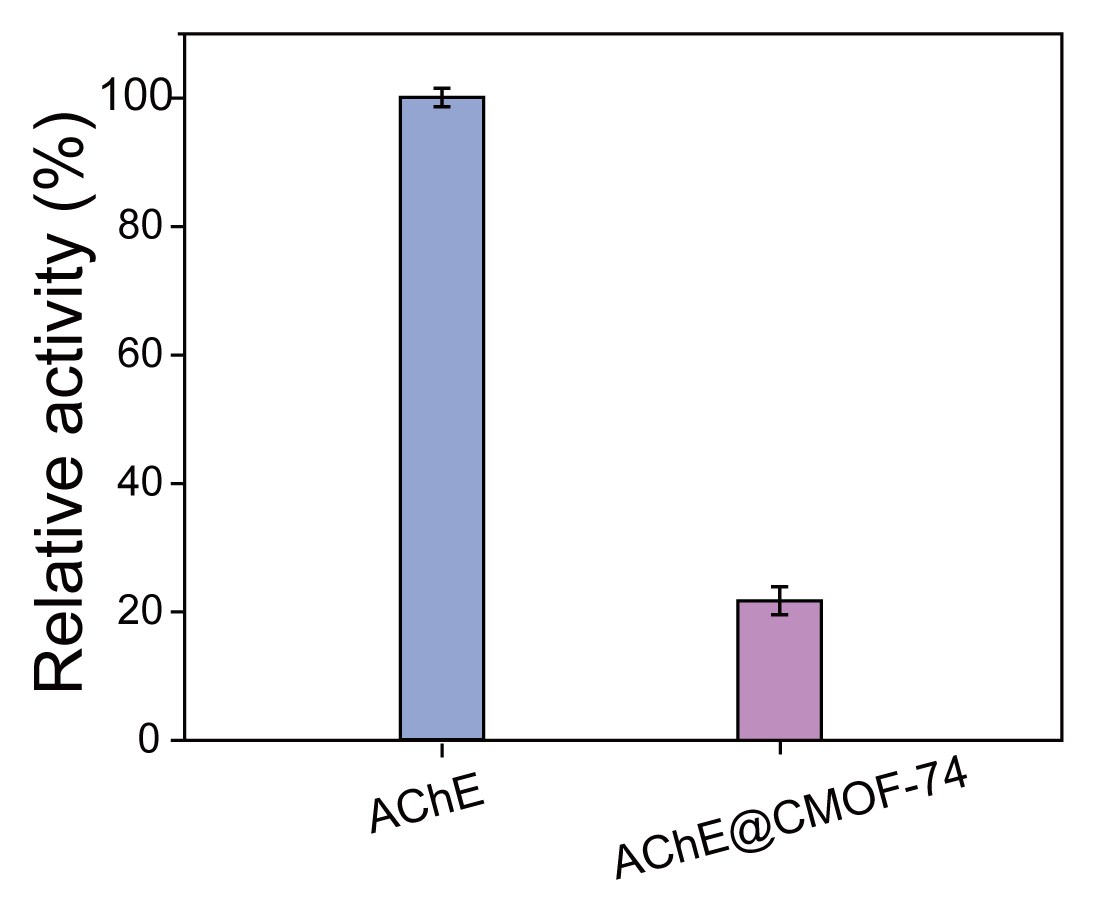
**

**Fig. S1** The relative activity (%) of AChE and AChE@CMOF-74 (the activity of free AChE is defined as 100%).

**
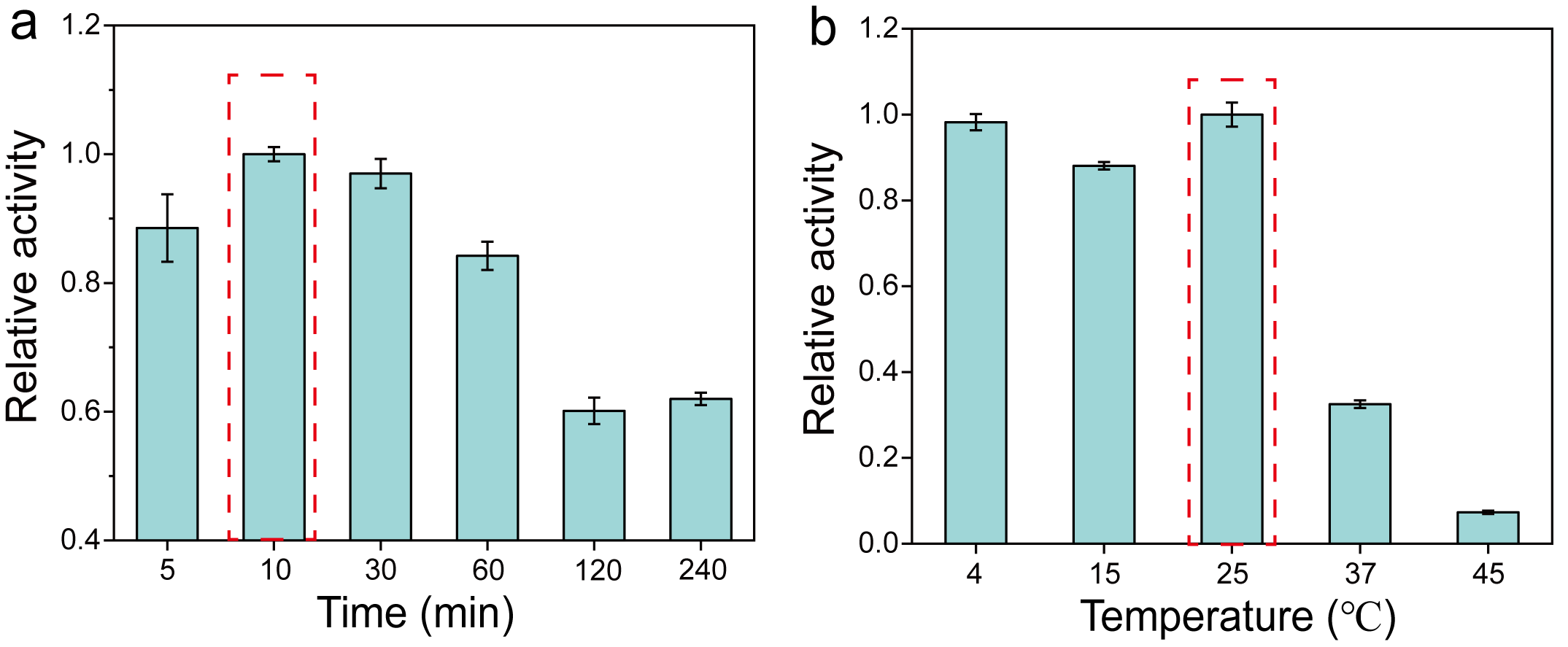
**

**Fig. S2** (a) The relative activity of AChE@AMOF-74 at different synthesis times. (b) The relative activity of AChE@AMOF-74 at different synthesis temperatures.

The synthesis temperature of 25 ℃ was selected primarily for practical and methodological considerations. Specifically, 25 ℃ corresponds to standard ambient laboratory conditions, which enables facile synthesis using a simple thermos tatted water bath without the need for specialized low-temperature equipment. In contrast, synthesis at 4 °C would require continuous and precise temperature regulation (e.g., using a refrigerated incubator), which could reduce operational simplicity and scalability. Therefore, the use of 25 ℃ ensures the simplest and most reproducible synthetic protocol without compromising the catalytic performance of AChE@AMOF-74.


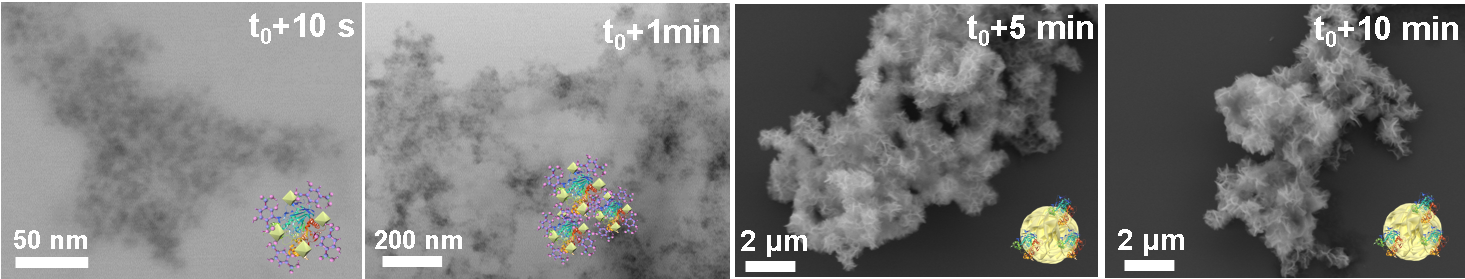


**Fig. S3** TEM and SEM images of AChE@AMOF-74 at different times.

To elucidate the formation mechanism of AChE@AMOF-74, the morphological evolution dynamics of the assembly were observed through TEM and SEM image **(Fig. S3).** The amorphous particles (average diameter of ≈ 6 nm) quickly appear within t_0_ + 10 s (t_0_ < 5 s). These particles display aggregate states when the reaction time lasts t_0_ + 1 min, corresponding element mapping images indicated that AChE is conducive to forming heterogeneous amorphous phases by fast aggregating and compete Zn^2+^ **(Fig. S4).** Afterward, the dense aggregate, which serves as a direct precursor of AChE@AMOF-74, transforms into amorphous nanoflower-like morphology nanostructures at t_0_ + 10 min. On the basis of the observations, we propose that the main mechanism for the formation of AChE@AMOF-74 can be divided into two stages: i) Amorphous aggregates are initially formed in the early stages of the reaction, influenced by the competition of AChE and Zn^2+^ aggregation. ii) Subsequent growth of these aggregates results in the formation of amorphous nanoflower AChE@AMOF-74 structures.


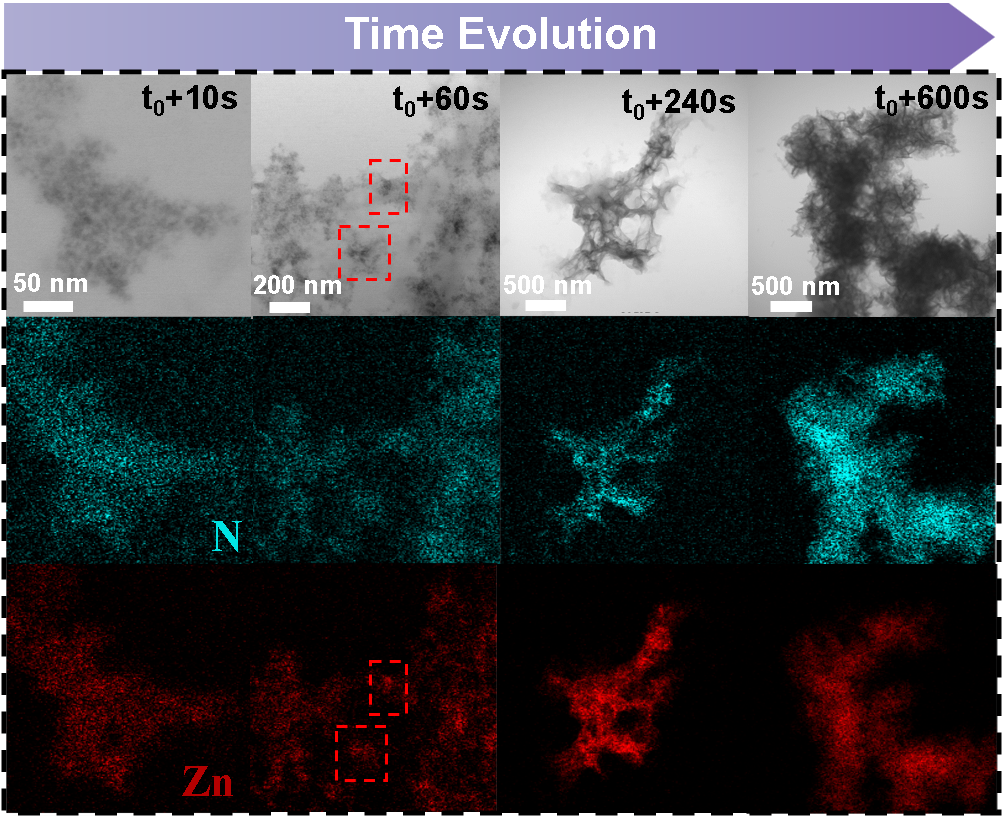


**Fig. S4** TEM and EDS mapping of AChE@MOF-74 at different times. The N element belongs to the AChE, and Zn is derived from MOF-74.


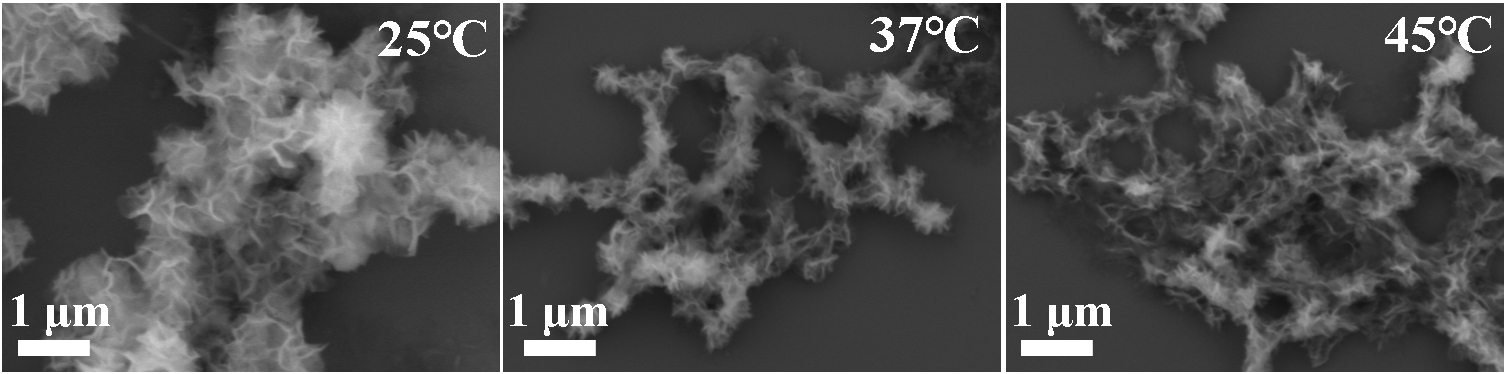


**Fig. S5** SEM images of AChE@AMOF-74 under different synthesis temperature.

we tested the variation in catalytic activity of AChE@AMOF-74 under different synthesis temperature conditions. The results show that when the synthesis temperature is below 25°C, the composite maintains high and consistent activity **(Fig. S2)**. However, as the temperature increases further, the activity of AChE@AMOF-74 significantly decreases, likely due to the unfavorable effect of higher temperatures on the formation of AChE@AMOF-74 **(Fig. S5)**.

**
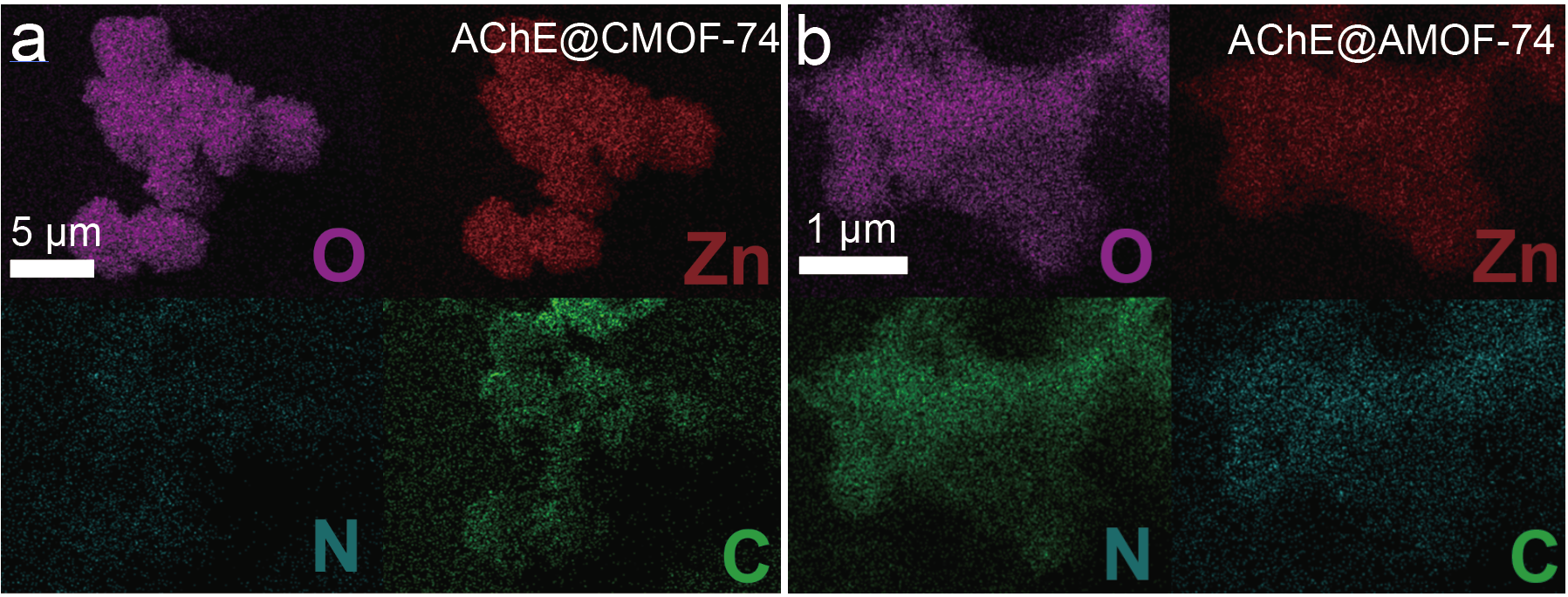
**

**Fig. S6** EDS mapping of (a) AChE@CMOF-74 and (b) AChE@AMOF-74, where N originates from AChE and Zn originates from the MOF-74 framework.

**
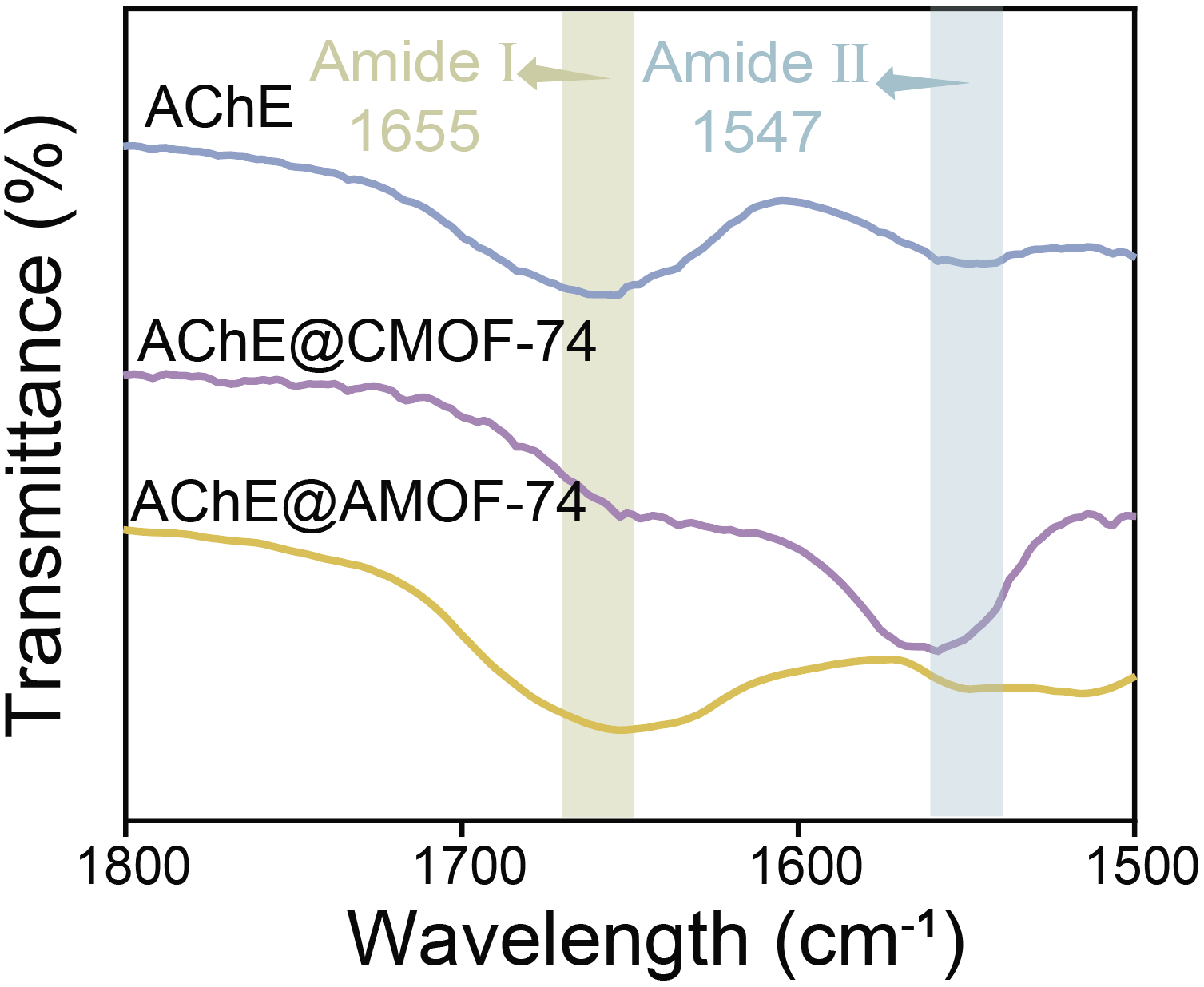
**

**Fig. S7** FT-IR spectra of AChE, AChE@AMOF-74 and AChE@CMOF-74 in the wavelength range of 1800-1500 cm^-1^.


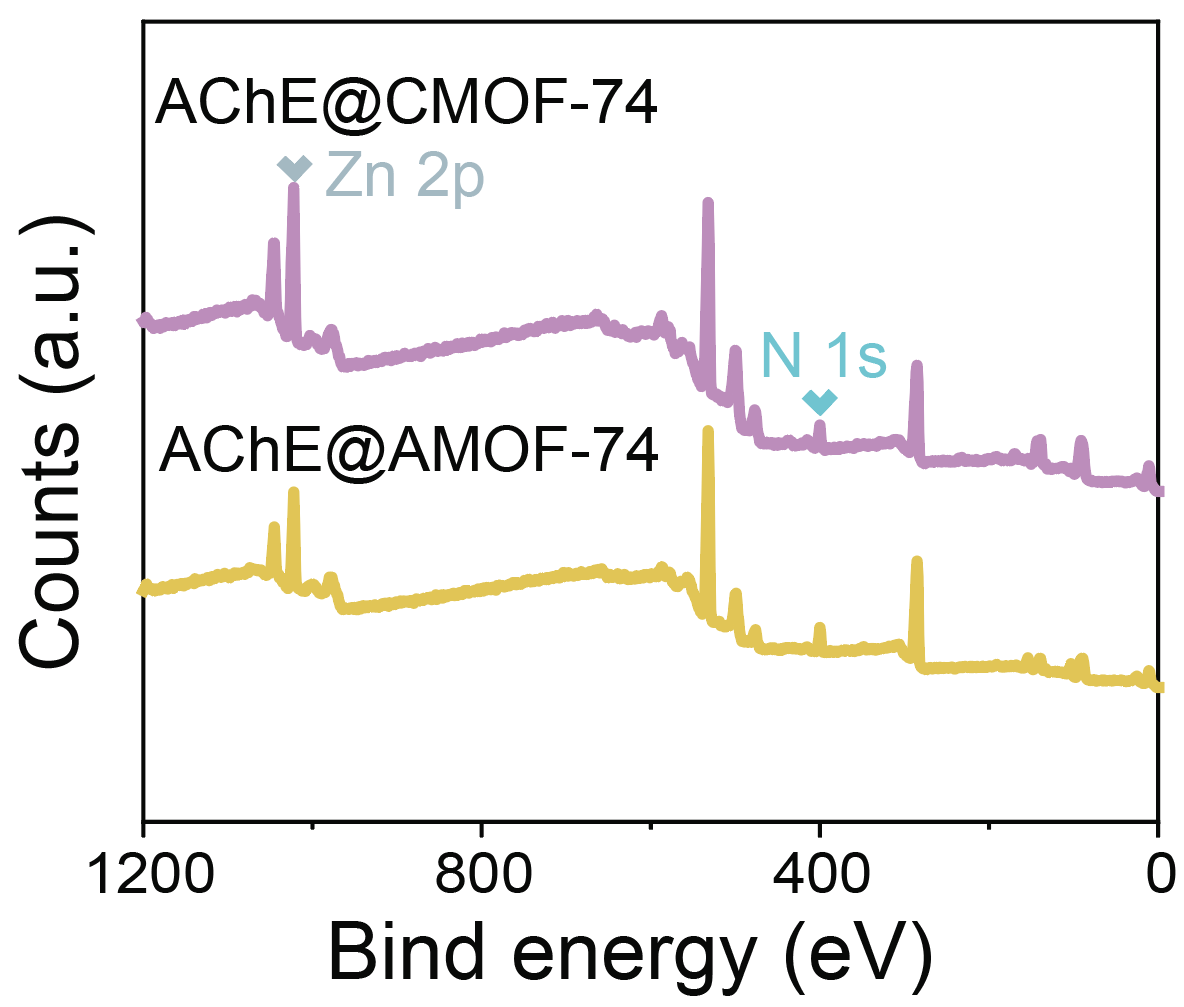


**Fig. S8** XPS spectra of AChE@CMOF-74 and AChE@AMOF-74.

**
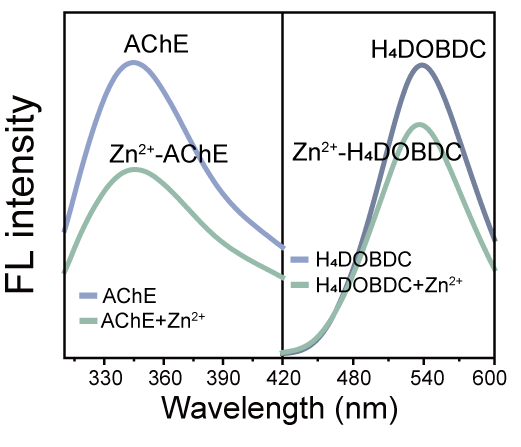
**

**Fig. S9** Fluorescence spectroscopy analysis of interactions between Zn²⁺ and AChE/H_4_DOBDC.

Deep analysis of the interactions between AChE, H_4_DOBDC and Zn²⁺ is necessary to elucidate the mechanisms behind these structural transitions. We introduced Zn²⁺ and observed a decrease in fluorescence for both AChE and H_4_DOBDC, confirming the successful formation of Zn²⁺-AChE and Zn²⁺-H_4_DOBDC complexes.


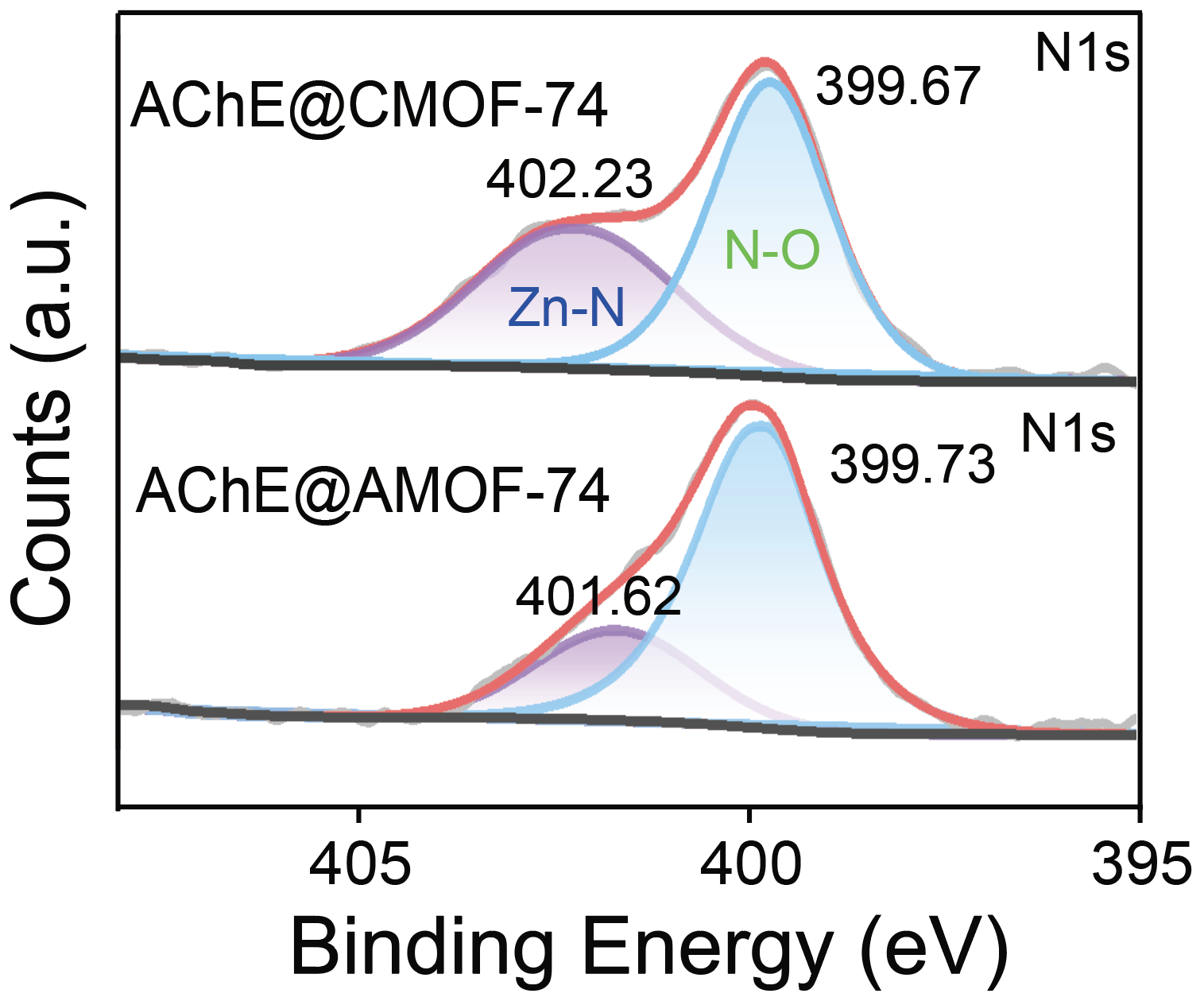


**Fig. S10** High-Resolution XPS spectra of N 1s of AChE@AMOF-74 and AChE@CMOF-74.


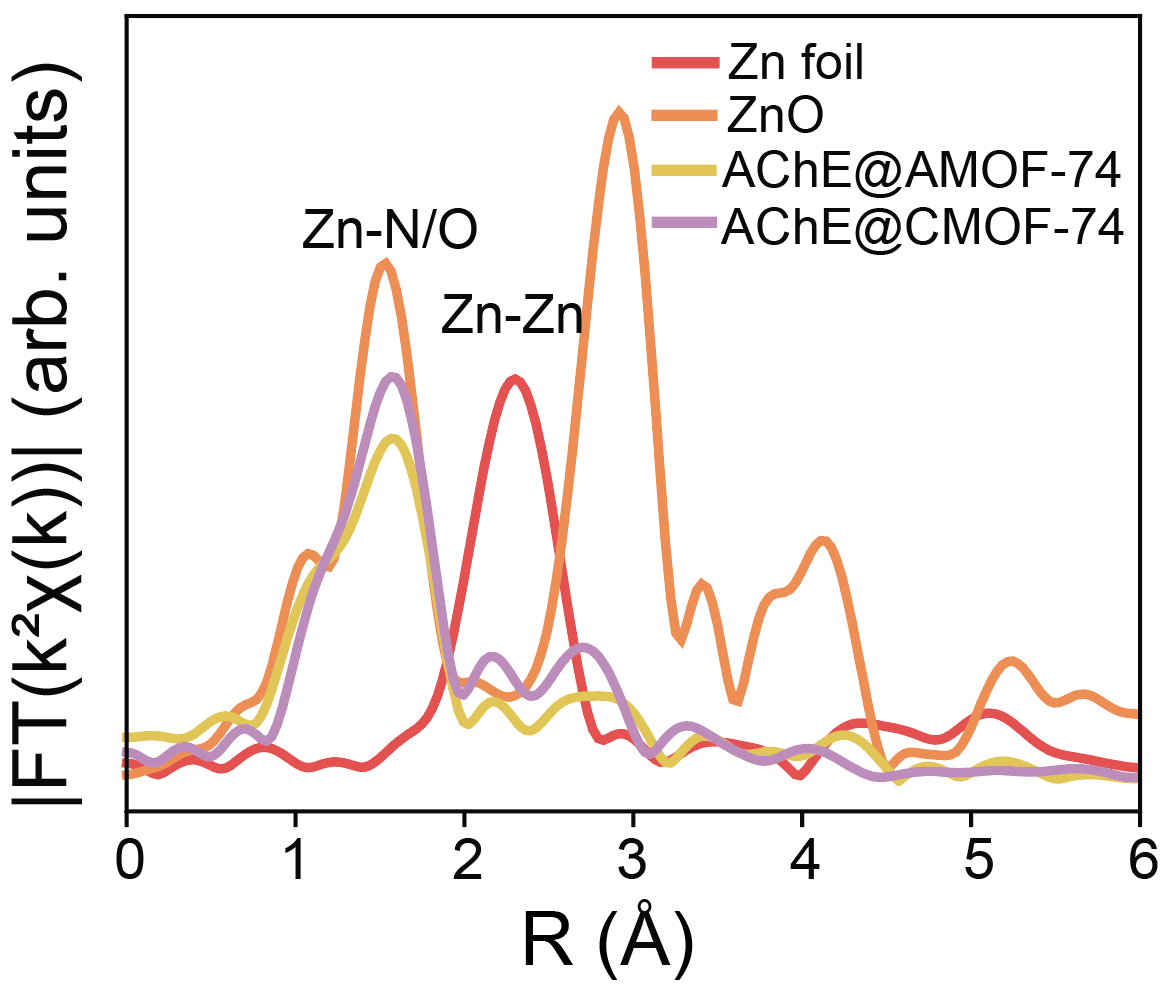


**Fig. S11** Fourier transforms of k^2^-weighted Zn K-edge EXAFS of AChE@AMOF-74, AChE@CMOF-74, ZnO and Zn-foil, respectively.


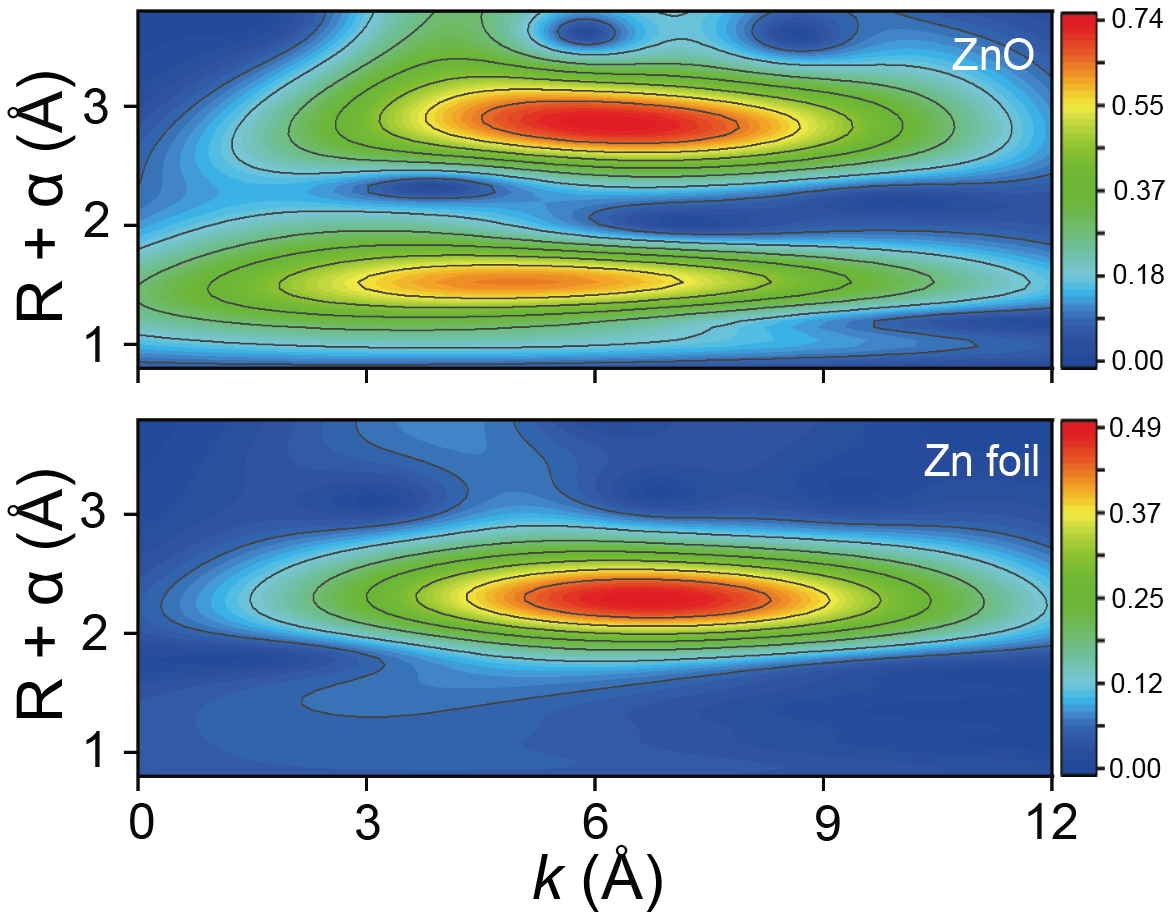


**Fig. S12** Wavelet transforms for the k^2^-weighted Zn K-edge of EXAFS signals of ZnO and Zn foil.


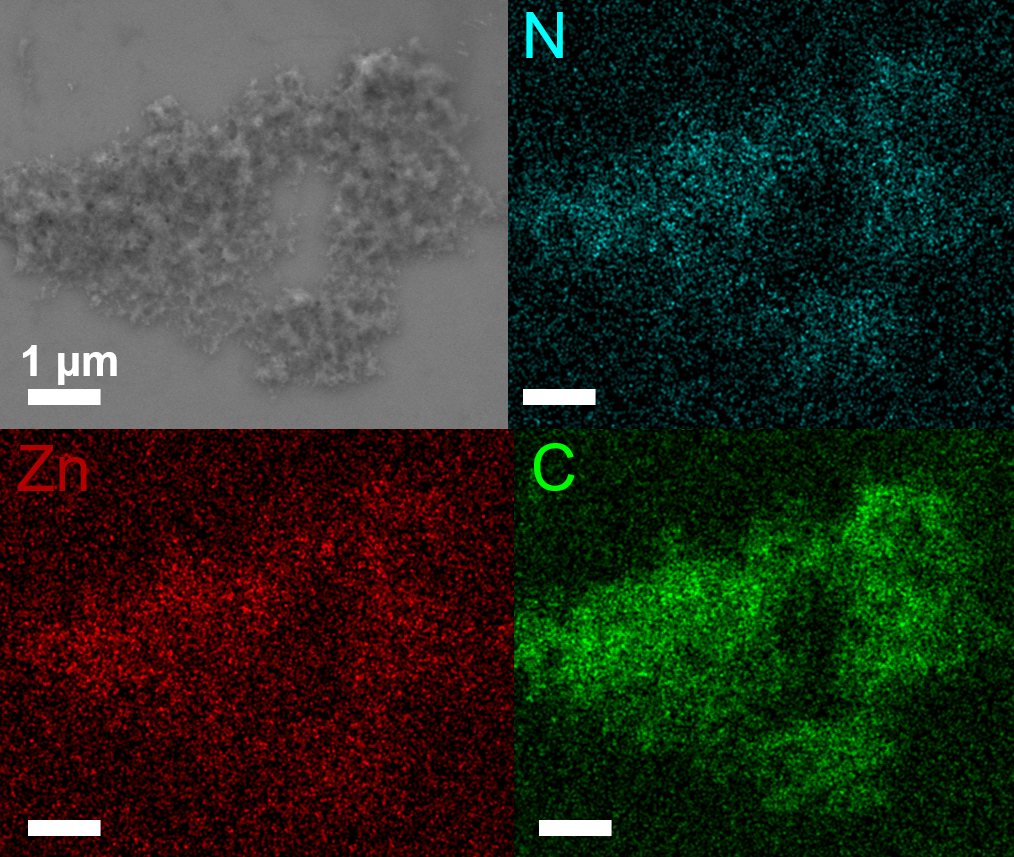


**Fig. S13** EDS mappings of AChE@MOF-74 at Zn^2+^=12.5 mM. The N element belongs to the AChE, and Zn is derived from MOF-74.

**
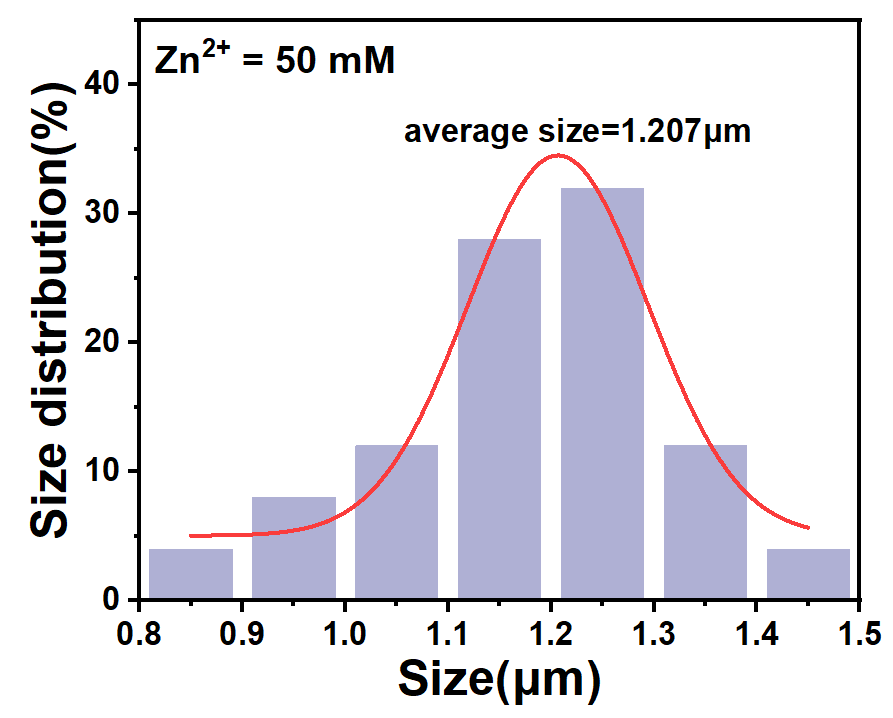
**

**Fig. S14** Size distribution of AChE@MOF-74 under Zn^2+^ concentration of 50 mM.

**
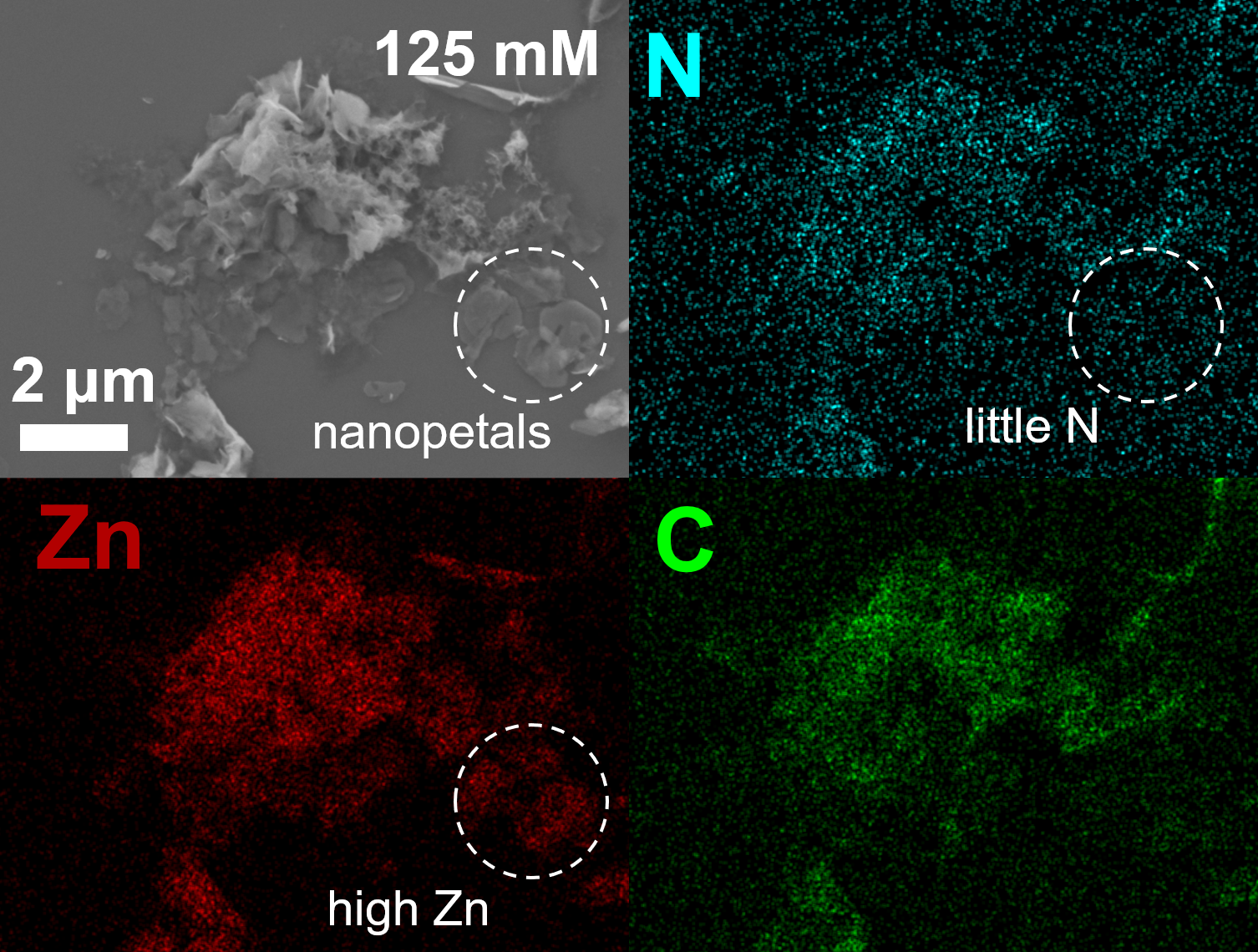
**

**Fig. S15** EDS mapping of AChE@MOF-74 (Zn²⁺ = 125 mM), where N originates from AChE and Zn from the MOF-74 framework.


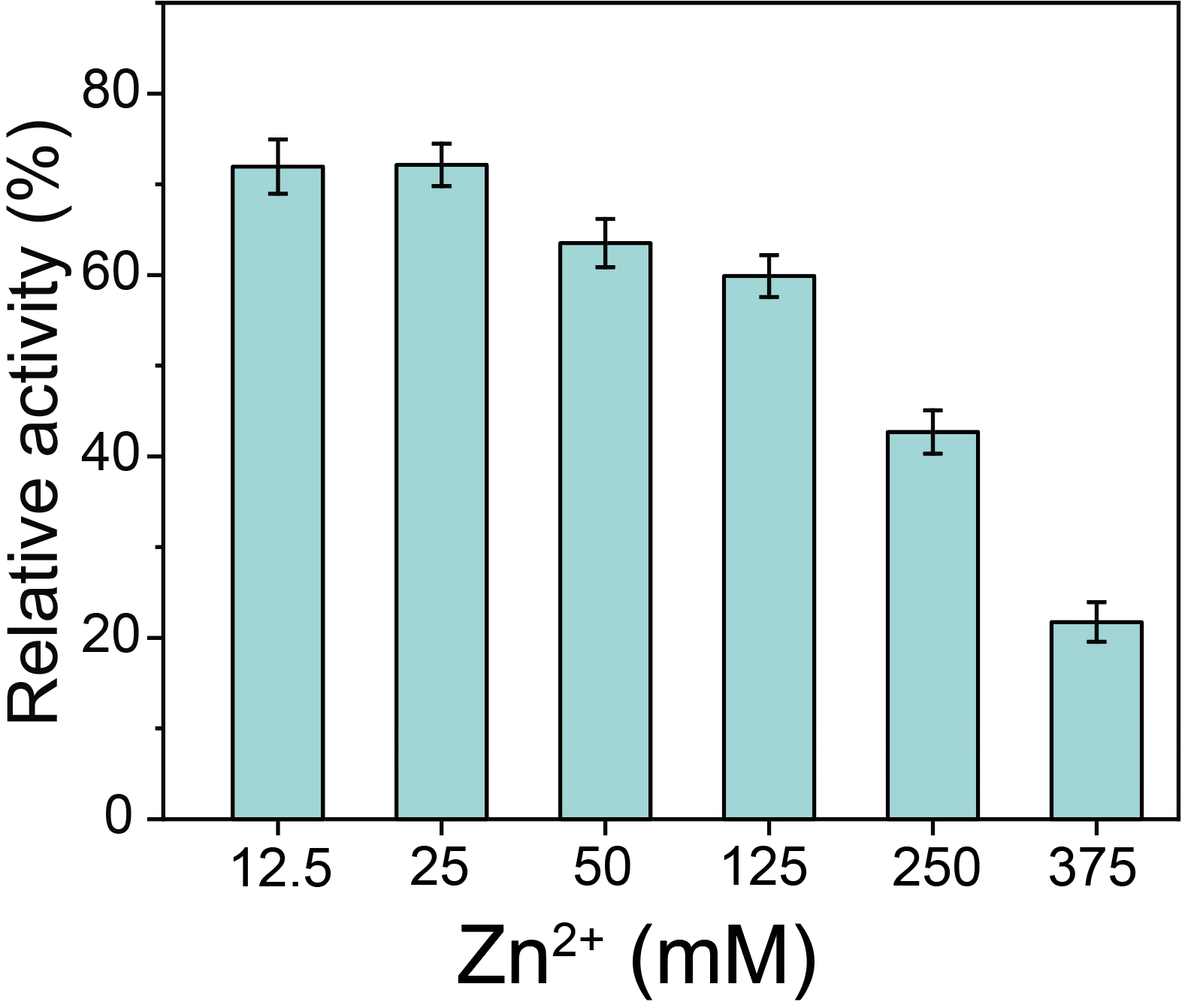


**Fig. S16** Relative activity (%) of AChE@MOF-74 under different Zn^2+^ concentrations.


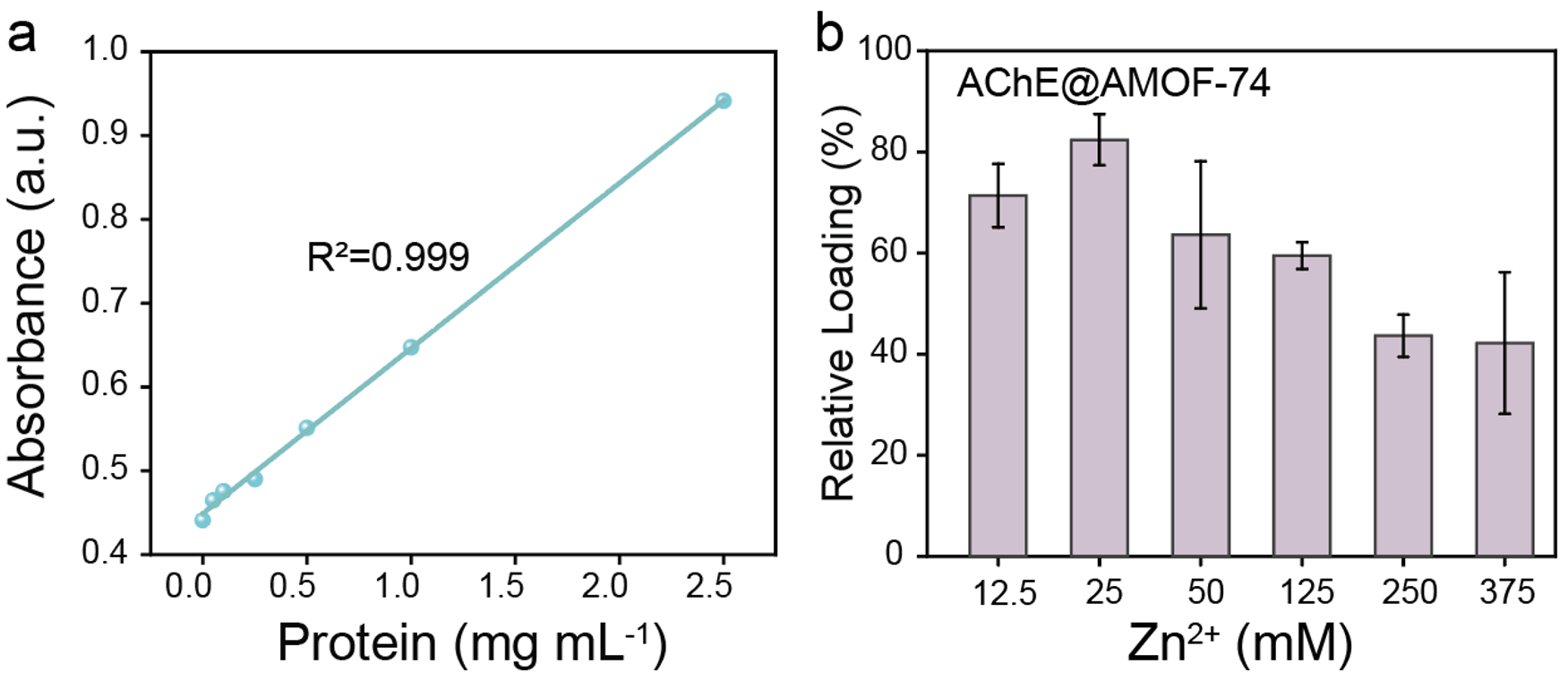


**Fig. S17** (a) Standard curve based on Bradford protein analysis. (b)Relative loading (%) of AChE@MOF-74 under different Zn^2+^ concentration (Zn^2+^ = 25 mM corresponds to AChE@AMOF-74; Zn^2+^ = 375 mM corresponds to AChE@CMOF-74).

We precisely regulated the defect density of the composites by adjusting the Zn²⁺ concentration, and quantitatively analyzed the enzyme loading of the resulting samples at different Zn^2+^ concentrations using the Bradford assay **(Fig. S17)**. The results show that AChE@AMOF-74 achieved a higher enzyme loading, which is consistent with its higher activity **(Fig. S16-S17)**. This can be attributed to the moderate defect density generated under 50 mM Zn^2+^, which provides enlarged pores and sufficient free space for enzyme encapsulation. In contrast, when the Zn^2+^ concentration is relatively high, the reduced number of coordination defects limits the available space for enzyme immobilization. Conversely, excessively low Zn²⁺ concentrations (25 mM) lead to the formation of amorphous aggregates that are unfavorable for enzyme loading **(Fig. S13)**. These results collectively indicate that a balanced defect level is crucial for achieving optimal enzyme loading, thereby supporting the selection of 50 mM Zn²⁺ as the optimal condition for synthesizing AChE@AMOF-74 with maximal catalytic performance.


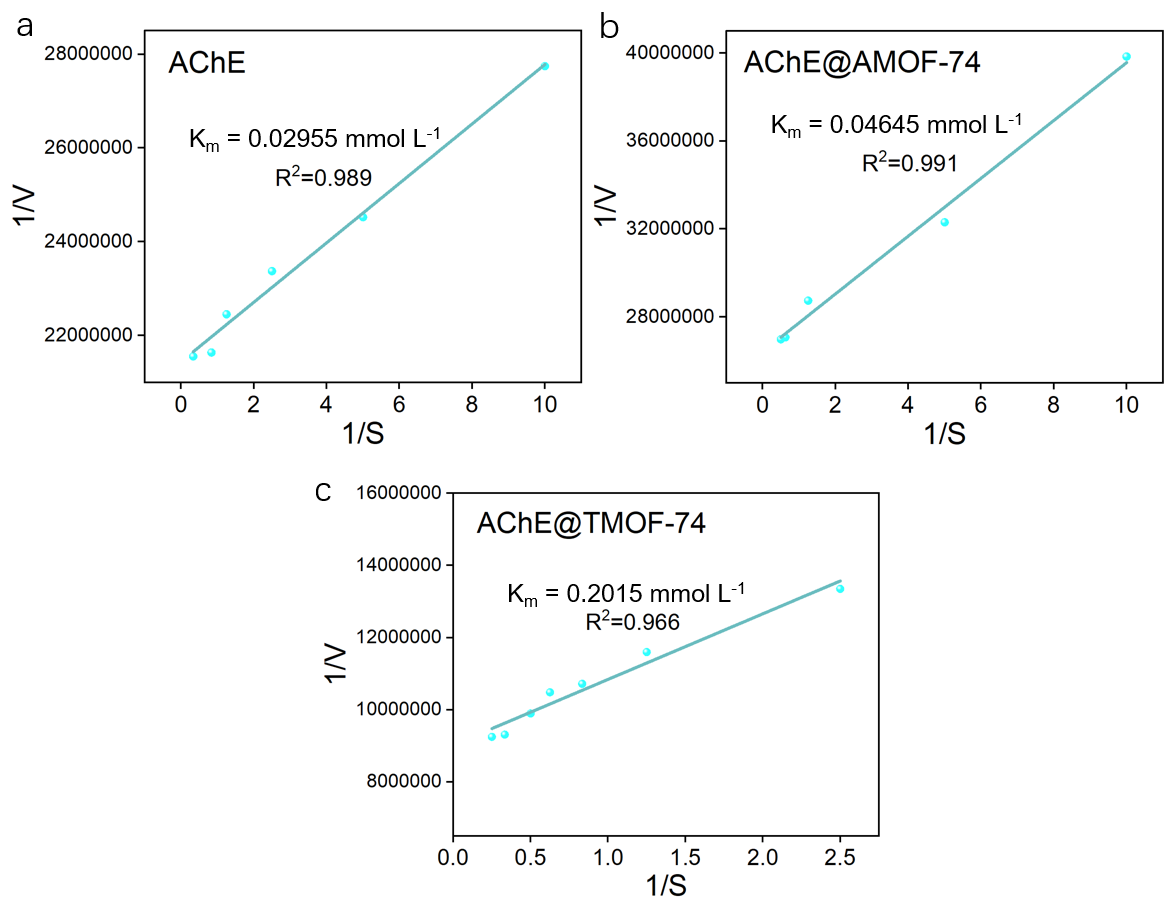


**Fig. S18** Lineweaver–Burke plots of (a) AChE, (b) AChE@AMOF-74 and (c) AChE@TMOF-74.


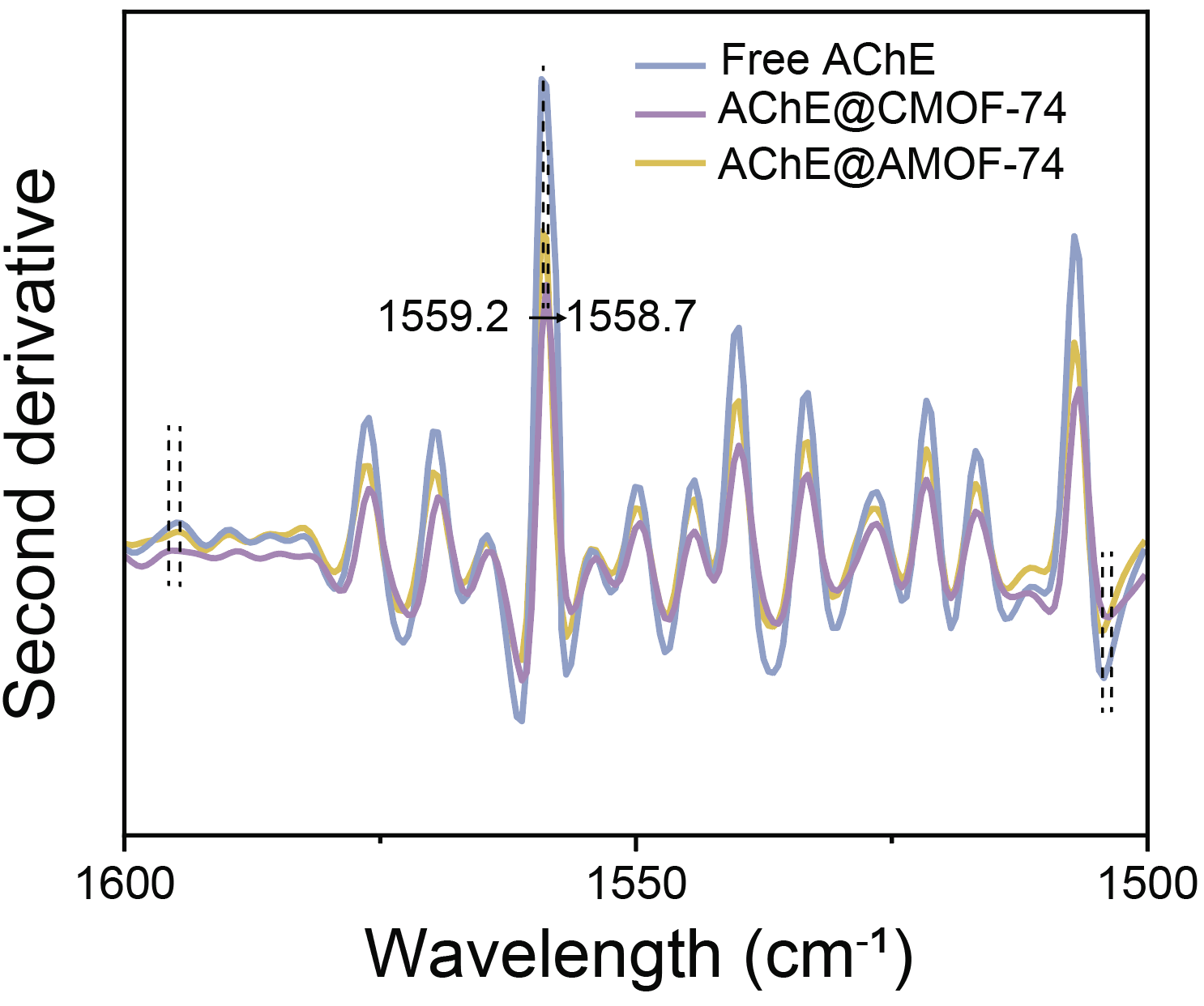


**Fig. S19** Second derivative FT-IR spectra of AChE, AChE@AMOF-74 and AChE@CMOF-74.

Careful observation of the second-derivative FT-IR spectra indicates that Zn–AChE interactions induce significant local perturbations at multiple sites. These interactions synergistically modify the local microenvironment of the enzyme, partially perturb its secondary structure, and consequently modulate the α-helix content in AChE@CMOF-74.

**
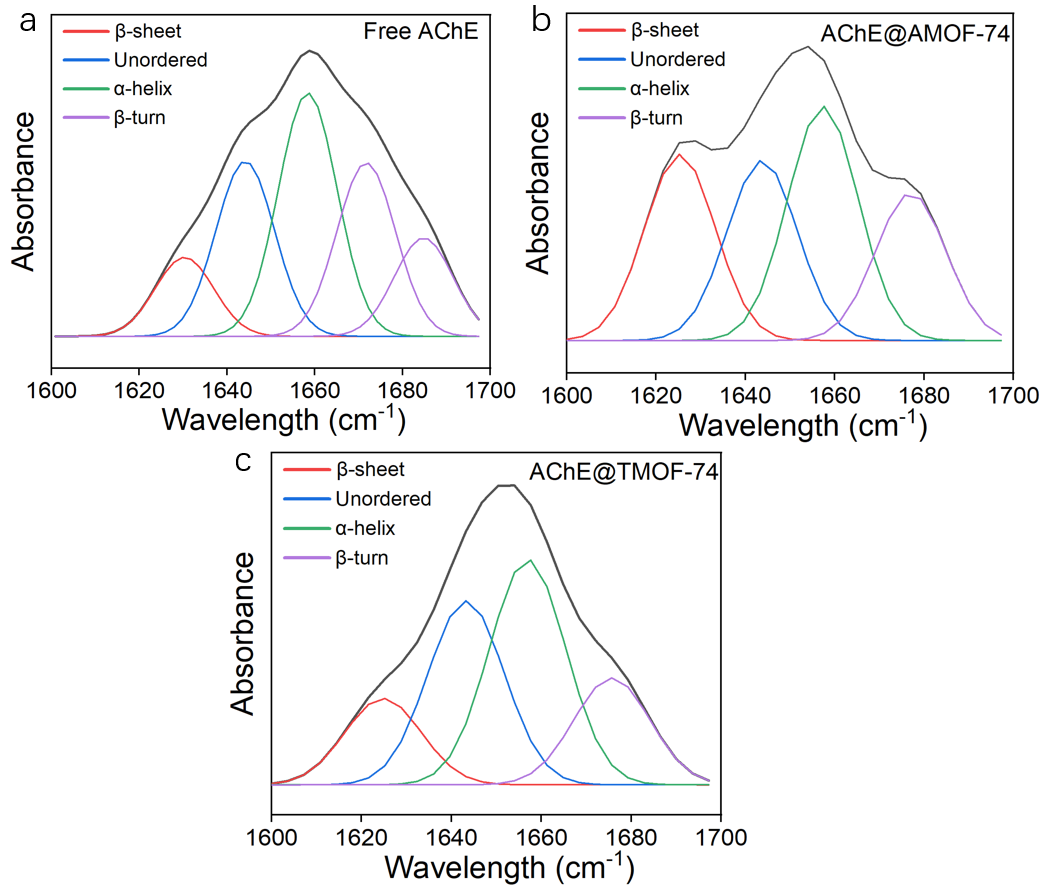
**

**Fig. S20** The structural contents of β-sheet, random coil structures, α-helix and β-turn were determined by Gaussian multi-component fitting. a) The secondary structure of free AChE (b-c) Secondary structure of AChE@AMOF-74 and AChE@TMOF-74

**
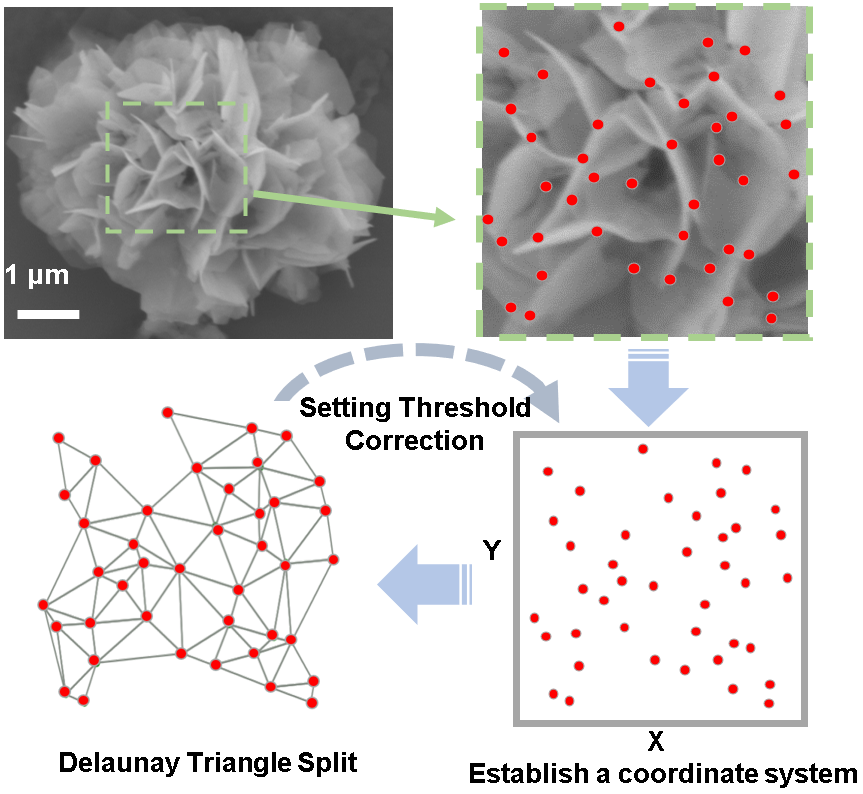
**

**Fig. S21** Flowchart for the statistical analysis of point spacing based on the Delaunay triangle partition method.

The SEM images were processed using Origin 2021 software. The pixel located at the lower-left corner of each SEM image was set as the origin. The images were digitized to obtain the coordinates of various intersection points for AChE@MOF-74 under different Zn^2+^ concentrations. A coordinate system was then constructed based on these coordinates. After obtaining the coordinates of the selected points, the x-y coordinates of AChE@MOF-74 were used to perform Delaunay triangulation with the Delaunay method from the SciPy library in Python. This process established the connectivity between the points. Subsequently, the average length of all triangle edges was calculated **(Fig. S21)**.

**
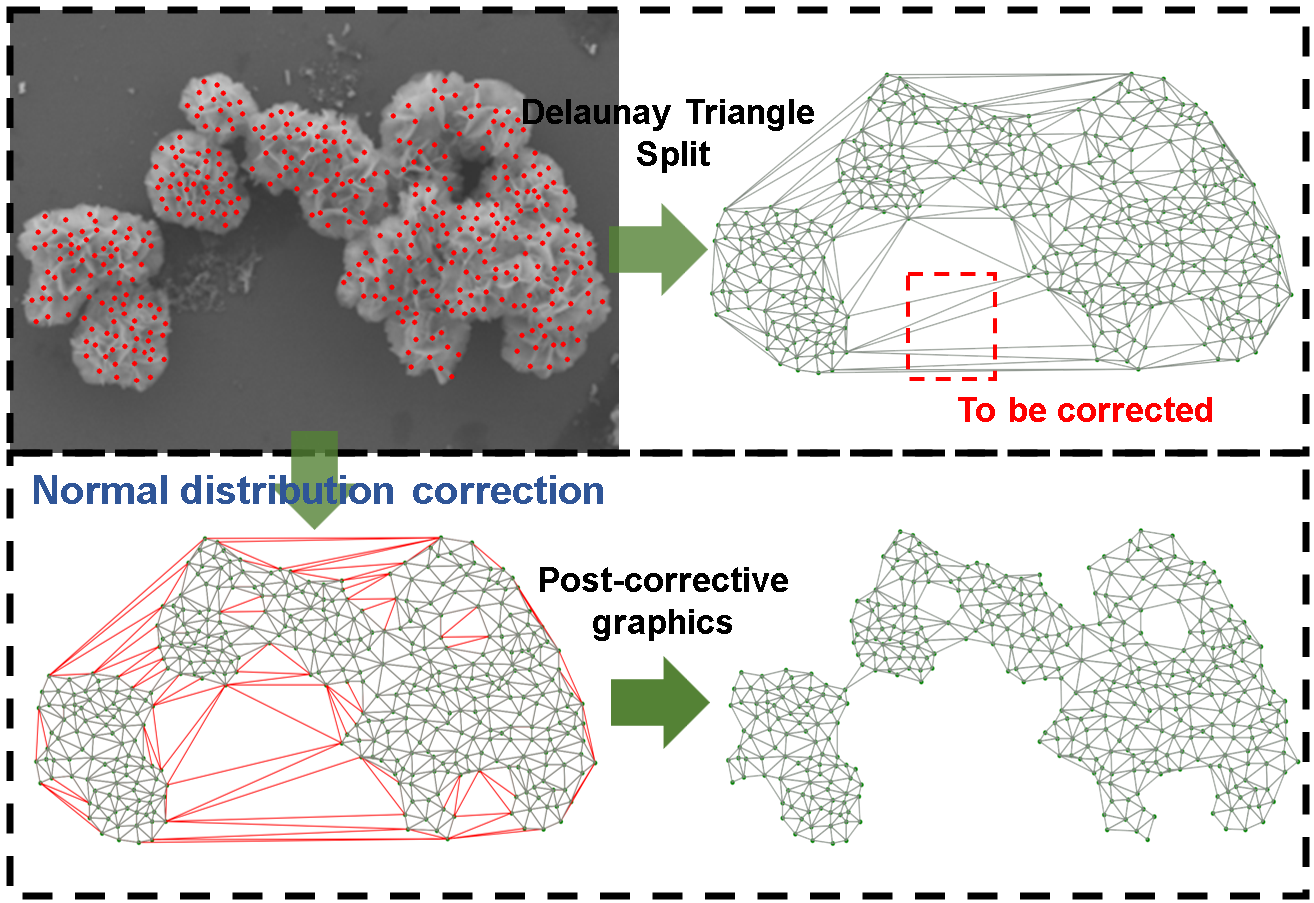
**

**Fig. S22** Normal distribution correction of Delaunay triangle partition in multi-AChE@MOF-74 particle ensembles (Confidence interval of 0.95).

In practical applications, boundary errors may arise, where initially non-adjacent points are incorrectly connected during the triangulation process. Therefore, it is essential that these erroneous connections be filtered out. To achieve this, the average edge length and variance of all edges within the set of triangle points were calculated, and a normal distribution model was established. The filtering threshold was set at the upper limit of the 95% confidence interval of the normal distribution: Z = 1.96. Specifically, edges with lengths less than μ (mean) + 1.96σ (standard deviation) were retained, while edges exceeding this length were discarded **(Fig. S22)**. The average length of the remaining edges was then recalculated and used as the final result.


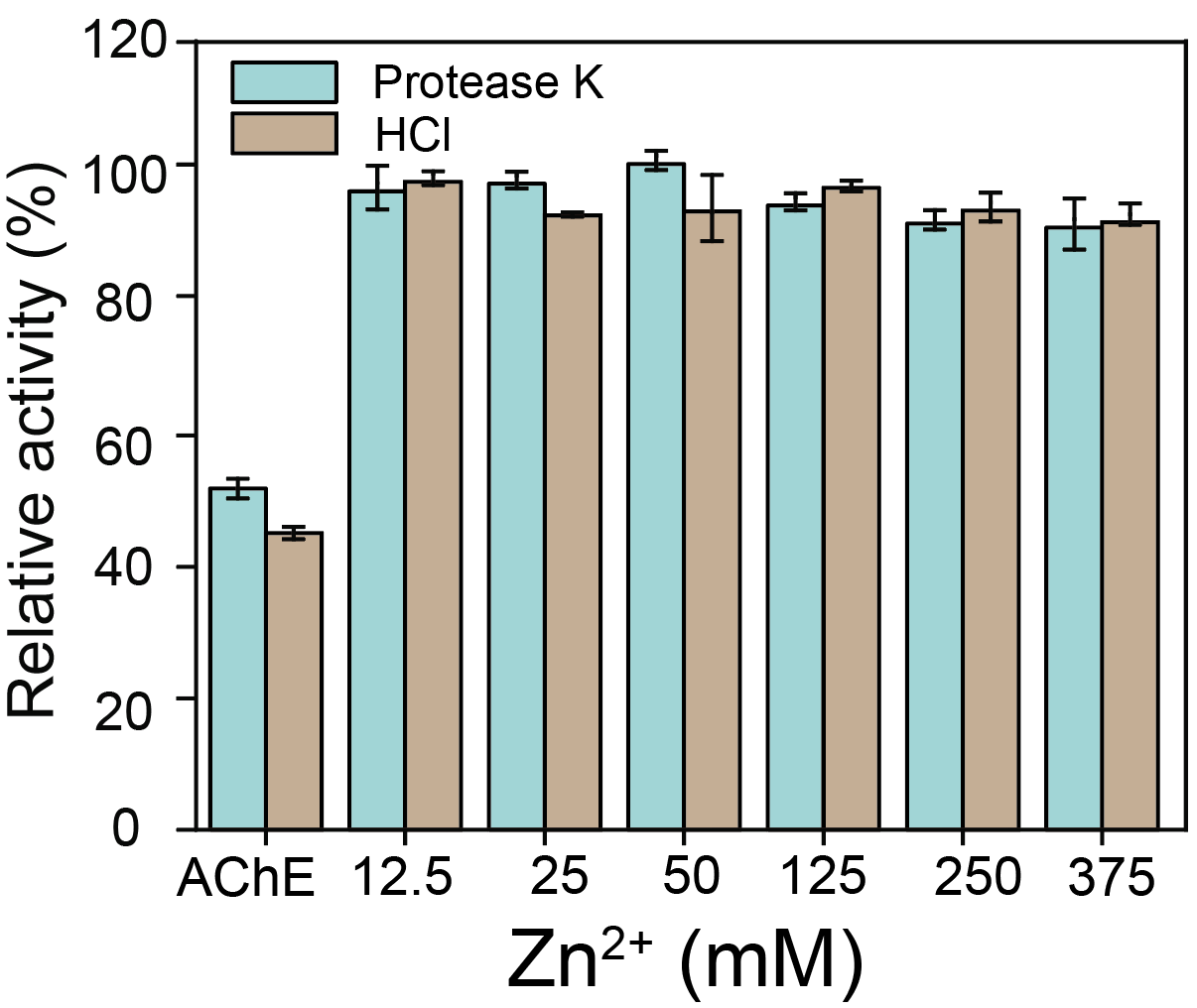


**Fig. S23** Stability of AChE@MOF-74 nanocomposites synthesized under different Zn^2+^ concentrations and free AChE after incubation with proteinase K (0.1 mg·mL^-1^) or HCl (0.01 M) for 30 min.


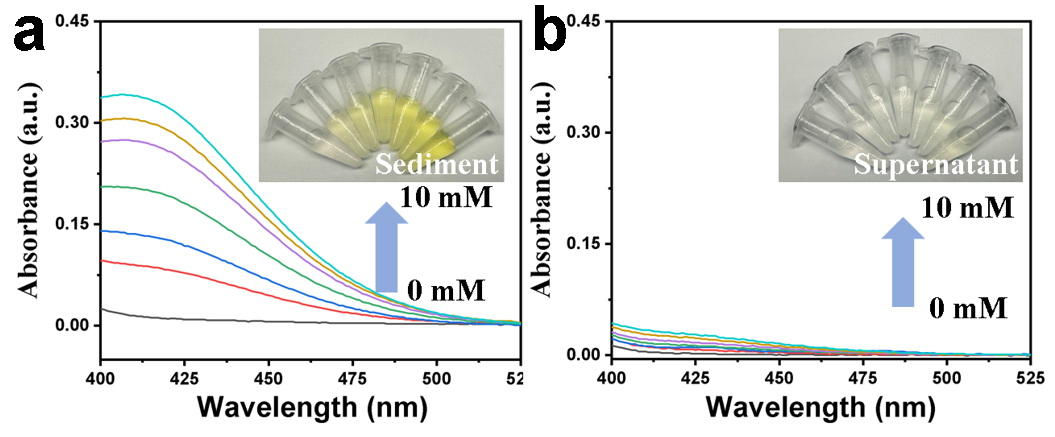


**Fig. S24** (a) Change in absorbance of AChE@AMOF-74 precipitation at different concentrations of substrate. (b) Change in absorbance of AChE@AMOF-74 supernatant at different concentrations of substrate.

During the synthesis of AChE@AMOF-74, we took the precipitate and supernatant after centrifugation and let them react with different concentrations of substrates, and then added DTNB and measured the UV spectrum of the system. The color change of the precipitate and supernatant confirmed that the enzyme was successfully bound to MOF and was enzymatically.

**
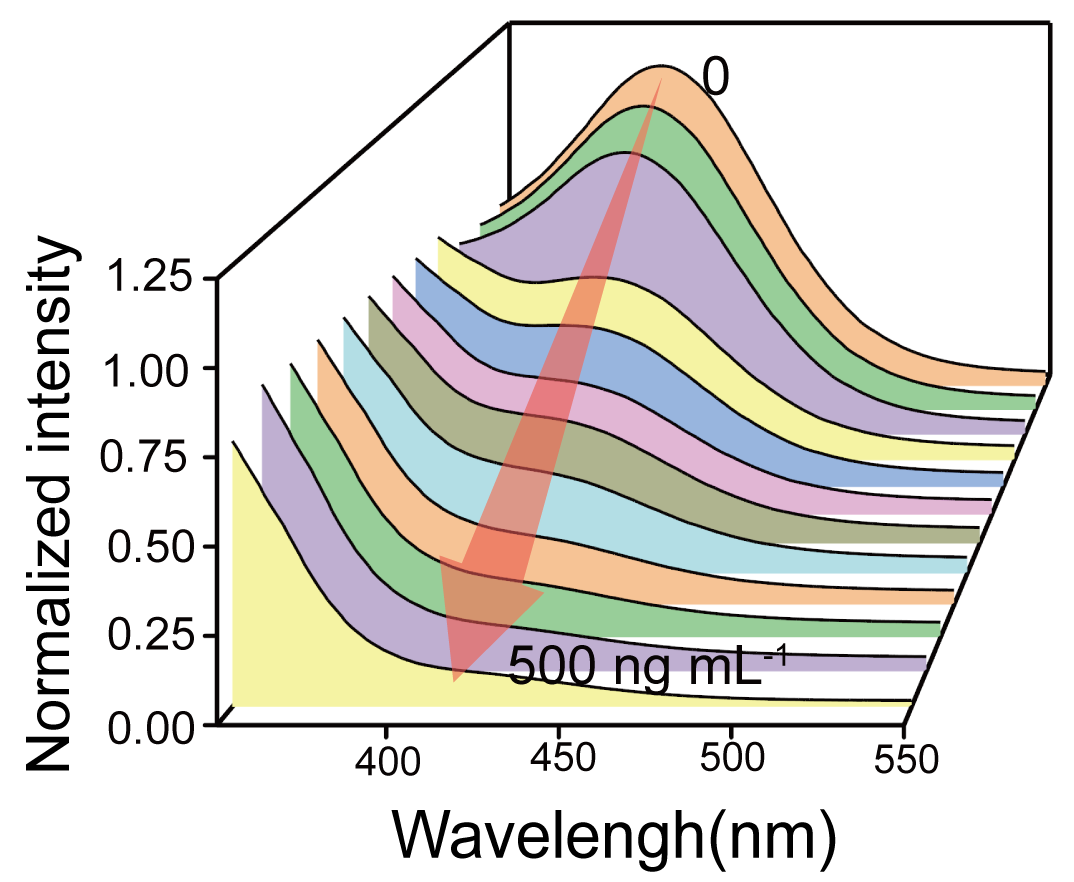
**

**Fig. S25** Normalized intensity of the AChE@AMOF-74 system under varying paraoxon concentrations (0-500 ng mL^-1^).

**
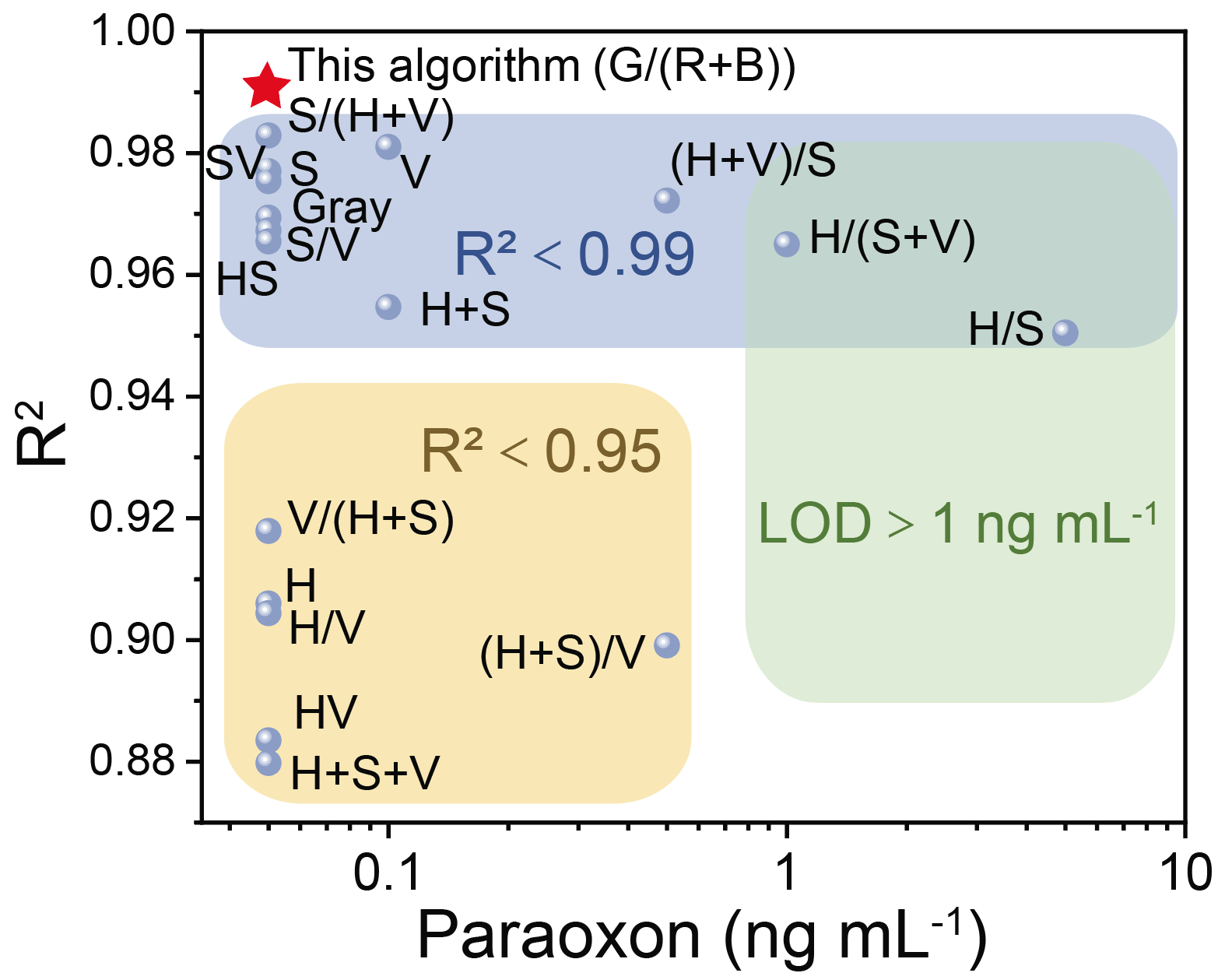
**

**Fig. S26** Comparison of the G/(R+B) algorithm with grayscale-based and HSV-based models.


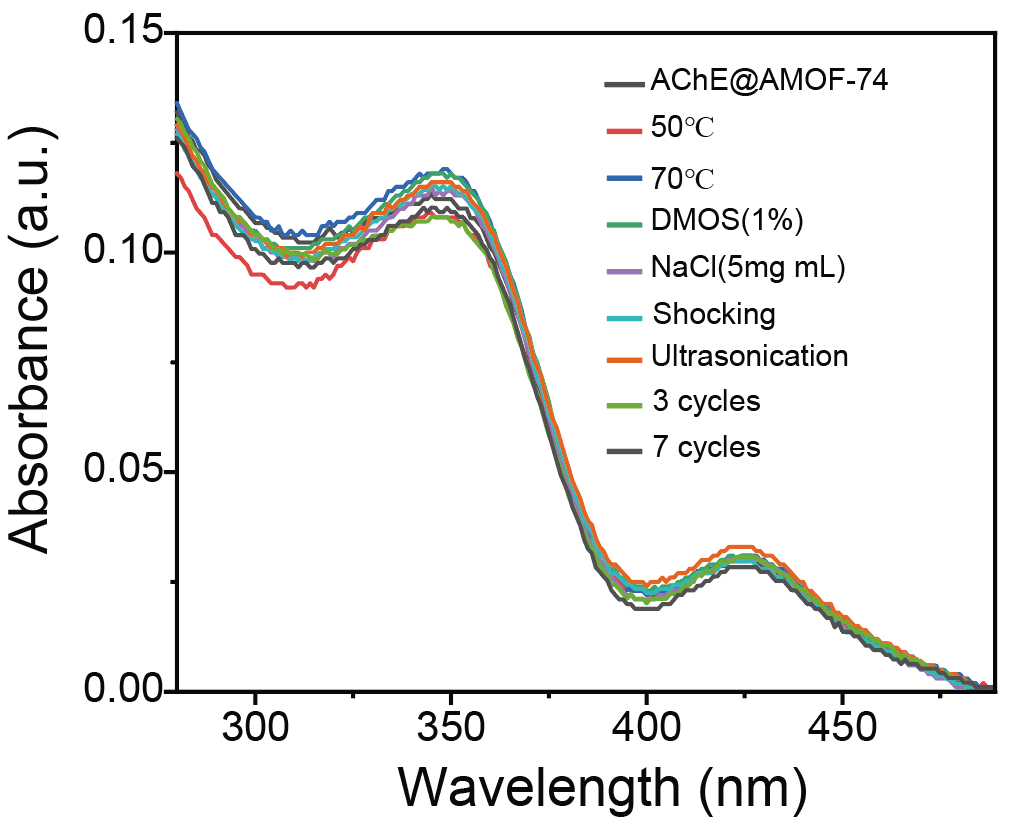


**Fig. S27** UV-vis absorption spectra of AChE@AMOF-74 after exposure to high temperature (50℃ and 70℃), chemical reagents (DMSO and NaCl), physical damage (Shocking and Ultrasonication), and repeated cycling tests (3 and 7 cycles).

**Table S1.** EXAFS fittings parameters at the Zn K-edge for various samples

| Sample | Shell | Bound length (Å) | CN | σ^2^ (Å^2^) | E0 shift  (eV) | R-factor |
| --- | --- | --- | --- | --- | --- | --- |
| AChE@CMOF-74 | Zn-N/O | 2.04±0.01 | 4.4±0.2 | 0.008±0.001 | -1.6±1.0 | 0.010 |
|  | Zn-Zn | 3.14±0.02 | 1.2±1.0 | 0.009±0.007 |  |  |
| AChE@AMOF-74 | Zn-N/O | 2.04±0.02 | 4.0±0.3 | 0.010±0.003 | -2.4±1.8 | 0.019 |

[a]. Bond length is the interactomic distance; [b]. CN is the coordination number; [c]. σ^2^ is Debye-Waller factor (a measure of thermal and static disorder in absorber scatter distance); [d]. E_0_  shift is edge-energy shift (the difference between the zero kinetic energy value of the sample and that of the theoretical model); [e]. R factor is used to value the goodness of the fitting.

**Table S2.** Comparison of detection limits and linearity of different image processing algorithms.

| Algorithms | LOD (ng mL^-1^) | R^2^ |
| --- | --- | --- |
| RG | 0.1 | 0.924 |
| RB | 0.05 | 0.975 |
| R/G | 0.5 | 0.946 |
| R/B | 0.5 | 0.9798 |
| R/(G+B) | 0.05 | 0.9803 |
| (R+G)/B | 0.5 | 0.981 |
| (R+B)/G | 0.5 | 0.964 |
| R+G | 0.1 | 0.978 |
| GB | 5 | 0.9794 |
| G/B | 0.5 | 0.982 |
| G/(R+B) | 0.05 | 0.9905 |
| B/(G+R) | 5 | 0.983 |
| B/G | 1 | 0.9623 |
| RGB | 0.05 | 0.9698 |
| R+G+B | 0.05 | 0.969 |
| RG/B | 0.5 | 0.981 |

**Table S3.** Comparison of the performance of different detection methods for OPs.

| Materials | Linear range  (ng mL^-1^) | Linear range width | LOD  (ng mL^-1^) | Ref |
| --- | --- | --- | --- | --- |
| 3D μPAD | 2.5-120 | 48 | 1 | ^[1]^ |
| ThT@ZnCPs | 5-1000 | 200 | 1.8 | ^[2]^ |
| MCM | 1-200 | 200 | 1 | ^[3]^ |
| Q-body | 16.21-848.81 | 52.36 | 5.09 | ^[4]^ |
| Escherichia coli BL21/pNP-LacZ | 25-7500 | 300 | 9.57 | ^[5]^ |
| PTDNP-MnNFs | 0.1-50 | 500 | 0.73 | ^[6]^ |
| Fe-Co MNPs | 5-5000 | 1000 | 1.67 | ^[7]^ |
| Mn-AIEMOF | 5-5000 | 1000 | 3.79 | ^[8]^ |
| Pt@ZIF-8@TMS | 1-500 | 500 | 0.7 | ^[9]^ |
| Cu@GDY | 5-200 | 40 | 1 | ^[10]^ |
| Ce2O2CN2/NC | 29.13-42022 | 1440 | 39.34 | ^[11]^ |
| Eu@Ce/UiO-67 | 145.65-58258 | 400 | 1.53 | ^[12]^ |
| AChE@AMOF-74 | 0.05-500 | 10000 | 0.05 | This work |

**References**

[1] X. Tong, G. Cai, L. Xie, T. Wang, Y. Zhu, Y. Peng, C. Tong, S. Shi and Y. Guo, Biosensors and Bioelectronics. **2022**, 222, 114981.

[2] Y. Li, Z. Huang, B. Liu, Z.-Z. Huang, H. Yang and H. Tan, Biosensors and Bioelectronics. **2022**, .220,114890.

[3] S.-H. Wen, H. Zhang, S. Yu, J. Ma, J.-J. Zhu and Y. Zhou, Journal of Hazardous Materials. **2024**, 480, 135791.

[4] J. Yu, K. Zhao, Z. Zhang, Y. Zhang, X. Zhang and H. Ren, Water Research. **2023**, 250, 121051.

[5] Z. Ma, Y. Li, C. Lu and M. Li, Journal of Hazardous Materials. **2023**, 457, 131725.

[6] J. Chen, X. Chen, J. Zhao, S. Liu and Z. Chi, Biosensors and Bioelectronics. **2020**, 170, 112668.

[7] Z. Shen, D. Xu, G. Wang, L. Geng, R. Xu, G. Wang, Y. Guo and X. Sun, Journal of Hazardous Materials. **2022**, 440, 129707.

[8] J. Tang, T. Cai, N. Li, Z. Chen, J. Liu and H. Yang, Journal of Hazardous Materials. **2025**, 491, 137964.

[9] B. Shen, Q. Wu, Y. Guo, J. Qin, H. Chen, Y. Yang, Z. Liu, L. Li, W. Li and C. Zhu, Advanced Functional Materials. **2024**, 35(8), 15854.

[10] K. Niu, Z. Zuo, X. Lu, L. Zou and J. Chen, Biosensors and Bioelectronics. **2022**, 205, 114111.

[11] C. Liu, X. Cao, X. Ma, Y. Wang, M. Zhang, J. Qiu, J. Chen and H. Xue, Small. **2025**, 21(13), 2411212.

[12] X. Liao, B. Li, L. Wang and Y. Chen, Small. **2025**, 25, 2409216.
